# Psychological Treatment of Exhaustion Due to Persistent Non-Traumatic Stress: A Scoping Review

**DOI:** 10.1007/s12529-023-10185-y

**Published:** 2023-06-12

**Authors:** Jakob Clason van de Leur, Filip Jovicic, Andreas Åhslund, Lance M. McCracken, Monica Buhrman

**Affiliations:** 1 Department of Psychology, Uppsala University, Box 1225, 751 42 Uppsala, Sweden; 2 PBM Globen Rehab, Arenavägen 27, 121 77, Johanneshov, Sweden; 3 Capio Centrum För Smärta Och Utmattning, Krukmakargatan 37A, 118 51, Stockholm, Sweden

**Keywords:** Burnout, Exhaustion due to non-traumatic stress, Exhaustion disorder, Work-related depression, Clinical burnout, Process-based therapy

## Abstract

**Background:**

Exhaustion due to persistent non-traumatic stress (ENTS) is a significant health problem with substantial personal, social, and economic impact. While there are increasing studies of ENTS, there is no international agreement on how it should be diagnosed and treated. This scoping review aimed to map definitions, diagnoses, treatments, outcome measures, and outcomes in psychological treatment studies of ENTS. A further aim was to assess the quality of the treatments and map what change processes are described within ENTS interventions.

**Methods:**

A PRISMA-guided scoping review of psychological treatment studies delivered in a clinical setting for ENTS was conducted using the databases of PubMed, PsycINFO, and CINAHL.

**Results:**

Of the 60 studies included, the majority (87%) stemmed from Europe. The most recurrent term for ENTS was burnout, and the diagnosis most often utilized was exhaustion disorder. Several treatments were reported, the most frequent being cognitive behavioral therapy (CBT) (68%). Statistically significant outcomes relevant to ENTS were reported in 65% (*n* = 39) of the studies, with effect sizes between 0.13 and 1.80. In addition, 28% of the treatments were rated as high quality. The most frequent change processes described were dysfunctional sleep, avoidance, behavioral activation, irrational thoughts and beliefs, worry, perceived competence/positive management, psychological flexibility, and recuperation.

**Conclusions:**

While several treatments based on CBT show promising results for ENTS, there do not seem to be any uniformly established methods, theoretical models, or change processes. Instead of adopting a monocausal, syndromal, and potentially bio-reductionist perspective on ENTS, a process-based approach to treatment is encouraged.

**Supplementary Information:**

The online version contains supplementary material available at 10.1007/s12529-023-10185-y.

## Introduction

Persistent non-traumatic stress can have detrimental health effects and can result in symptoms of exhaustion, reduced performance, and cognitive impairments [1–4]. Today, stress‐related disorders are the leading cause of work absenteeism in the Organization for Economic Co-operation and Development (OECD), and the indirect costs of work‐related stress have been estimated to be €20 billion annually in the European Union [5–8]. A particular stress-related disorder called exhaustion due to persistent non-traumatic stress (ENTS) is an increasing subject of clinical research [9–14]. The cardinal symptoms of ENTS include substantial debilitating levels of exhaustion coupled with cognitive impairments, but not necessarily a depressed mood [15]. There is, however, no international agreement on if or how ENTS should be diagnosed and how to best treat it [16].

ENTS is not something new. In 1869, George Miller Beard described the diagnosis of neurasthenia, a condition — well known to clinical practitioners of that time — characterized by persistent exhaustion due to the fast pace and demands of the “modern life,” as Beard himself put it [17]. In the late nineteenth century, neurasthenia became one of the most common diagnoses used throughout the western world. The German psychiatrist Emil Kraepelin, known for his work with the classification of mental disorders, described neurasthenia as “the disease of our time” and distinguished it from other mental illnesses as an “acquired syndrome” [18]. However, with the introduction of psychoanalysis, favoring internal psychological explanations for mental disorders, followed by the world wars, the scientific interest in neurasthenia faded [19]. With the introduction of the non-etiological Diagnostic and Statistical Manual system (DSM) in psychiatry in the second half of the twentieth century, neurasthenia appears to have been subsumed within other diagnoses [20].

For the past 40 years, the interest in ENTS has been reawakened through the concept of burnout, a construct used in organizational psychology to describe how employees gradually succumb to exhaustion, cynicism, and professional inefficiency due to organizational factors [21]. Burnout is not classified as a disorder in the International Classification of Mental and Behavioral Disorders (ICD-10), nor in the Diagnostic and Statistical Manual, fifth edition (DSM-5) [22, 23]. Instead, *clinical burnout* is sometimes used to describe the end stage of a severe burnout process that requires clinical attention [24, 25]. In Sweden, the diagnosis of exhaustion disorder has been accepted into the Swedish ICD-10, and in other countries, terms such as work-related neurasthenia, “work-related depression,” “adjustment disorder,” and “somatization syndrome” are used interchangeably to describe clinical burnout/ENTS [15]. There is an ongoing debate as to whether ENTS should be considered a disorder on its own or whether it should be a part of depression, with diverging evidence currently supporting both positions [26–32]. The nosological confusion surrounding ENTS is matched with equal confusion around treatment, perhaps not surprisingly. No evidence-based treatment has been identified for ENTS, and it appears that various treatments with varying theoretical frameworks, components, and methods are utilized in regular care [16].

In recent years, there has been a call for psychological treatment research to increase its focus on treatment models (a) built upon testable theories, (b) targeting specific evidence-based processes of change, and (c) tailored to individual people’s needs [33]. Change processes are the specific variables through which a treatment method exerts its influence over a targeted dependent variable [34]. It is argued that these processes should be theory-based, manipulable, dynamic, and multilevel [35]. A focus on evidence-based change processes can have advantages compared to a focus on treatment types. Perhaps ENTS is not best understood as a discrete disease needing a specific treatment type. Maybe it is a condition that emerges from a network of pathological interactions of behavioral, cognitive, emotional, physiological, and social elements — a network that can be altered by targeting explicit processes of change, in specific situations, for particular people [35].

There is a clear need to assess existing clinical treatments for ENTS to help focus future theory development and increase international consensus. A scoping review is an excellent way to map a field of study particularly one still in its youth, where more detailed questions are not yet possible [36]. Therefore, the purpose of this scoping review was to map the different definitions, diagnoses, treatments, outcome measures, and outcomes achieved in clinical treatment studies of ENTS. In addition, given the increased focus on testable theories and processes of change within contemporary psychological treatment research, the current review also aimed to assess the quality of the treatments utilized for ENTS and explore what processes of change are described within these interventions.

## Method

This scoping review was conducted following the guidelines from the Joanna Briggs Institute, and the reporting follows the PRISMA-ScR guidelines [37, 38]. An unpublished protocol stating the scoping review process was developed a priori. Furthermore, a PRISMA-ScR checklist is supplied within the supplementary material.

### Information Sources and Database Search

Three databases, PubMed, PsycINFO, and CINAHL, were selected for the current scoping review. The research team developed search terms aimed at providing both scope and precision for each of the specific concepts included in this review: (a) ENTS and (b) psychological treatments or multimodal interventions with a substantial psychological component. First, a preliminary search was conducted. The team then added search terms to the list, including new search terms found in the search results and adjustments to accommodate each database.

The final search strategies for each database were discussed with two research librarians before commencing the search on January 7, 2021. The database search was repeated on May 10, 2022, to include recently published papers. In addition, the search was supplemented by reviewing the reference lists of the included articles.

### Eligibility Criteria

A study was eligible for inclusion if it (a) appeared as a full-text primary research report available in English; (b) included adult (≥ 18 years) participants; (c) included participants with conditions associated with exhaustion, including burnout, work-related depression, adjustment disorder, exhaustion disorder, due to some form of explicitly defined long term non-traumatic stressor; (d) involved treatment delivered in a clinical setting; (e) used a quantitative treatment design, including a clinical outcome; and (f) described or examined the impact of psychological treatment(s) or multimodal interventions with a substantial psychological component. Treatment was deemed psychological if it was based on psychotherapeutic or behavioral change principles and included related methods.

A study was excluded if it (a) targeted stress in general (rather than conditions associated with the presence of long-term stressors as described in the inclusion criteria), (b) if the severity of the condition and/or need for intervention by professionals in a clinical setting was not assessed or reported, (c) described treatment that was delivered in a non-clinical setting (e.g., workplace interventions), (d) solely used a qualitative study design, or (e) was a study protocol of a future study.

### Study Selection and Data Extraction

#### Screening

Before beginning the screening of abstracts, the complete search of each database was imported to Endnote, where duplicates were removed. Next, a pilot screening was carried out to ensure inter-rater reliability. Two of the reviewers independently applied the eligibility criteria to the random sample of 10 abstracts and compared their results (AÅ and JCvdL in the first search, JCvdL, and FJ in the updated search). There was an > 90% agreement in the results. The screening was then carried out independently, using the web-based tool Rayyan (https://rayyan.qcri.org/). If there was any uncertainty about whether a paper was eligible, the paper was included for a closer assessment in the selection phase.

#### Selection

Before commencing the selection process, another pilot selection of 10 articles was carried out to ensure inter-rater reliability with > 90% agreement (AÅ and JCvdL in the first search, JCvdL, and FJ in the updated search). The remaining full-text articles were independently read in full by three reviewers and assessed for eligibility (AÅ and JCvdL in the first search, JCvdL, and FJ in the updated search). Discrepancies were resolved by the fourth and fifth reviewers (LM, MB).

#### Data Extraction Process

In the extraction phase, three reviewers extracted information from all included papers into tables (JCvdL, AÅ, FJ). The following information was extracted: study location, design, number of participants, age, gender, control condition, setting, population, inclusion and exclusion criteria, terms used for ENTS, diagnostic label(s) used, type of intervention(s), treatment model used, suggested processes of change, profession delivering the intervention(s), the scope of the intervention(s), the format of intervention(s), use of follow-up measurement, outcome measures, outcome variables (primary and secondary), significant outcomes, and effect sizes. The average mean and SD were pooled in cases where age, means, and standard deviations (SD) were presented separately for different treatment conditions [39].

#### Quality Assessment

Based on the recommendations of Hayes et al. [40], a quality assessment tool was developed. A psychological treatment is of high quality if: *The intervention is rooted in a theoretical model that serves as an organizing framework for predicting psychological change with high clinical utility. The framework includes specific, quantifiable components and defines processes and outcomes (dependent and independent variables). These stipulated processes of change are based on scientifically well-established psychological principles*. Based on this definition, four criteria were extrapolated, yielding a treatment quality score ranging from zero to four:

- Is there an explicit theoretical framework described as the basis for treatment? (Yes = 1 point, No = 0 point)
- Is the theoretical framework based on scientifically well-established psychological principles? (Yes = 1 point, No = 0 point)
- Are processes of change described within the theoretical framework? (Yes = 1 point, No = 0 point)
- Are specific methods or components targeting the proposed processes of change described? (Yes = 1 point, No = 0 point)

A pilot treatment quality assessment was implemented once more to ensure inter-rater reliability. First, one article was rated together by two reviewers (JCvdL and AÅ), followed by a pilot rating of five papers with > 90% agreement (4 ratings per paper, making it a total of 20 ratings). Then, all remaining papers were quality assessed independently, and discrepancies were resolved with the rest of the reviewers (LM, MB, FJ).

## Results

### Selection of Sources of Evidence

**Fig. 1:**
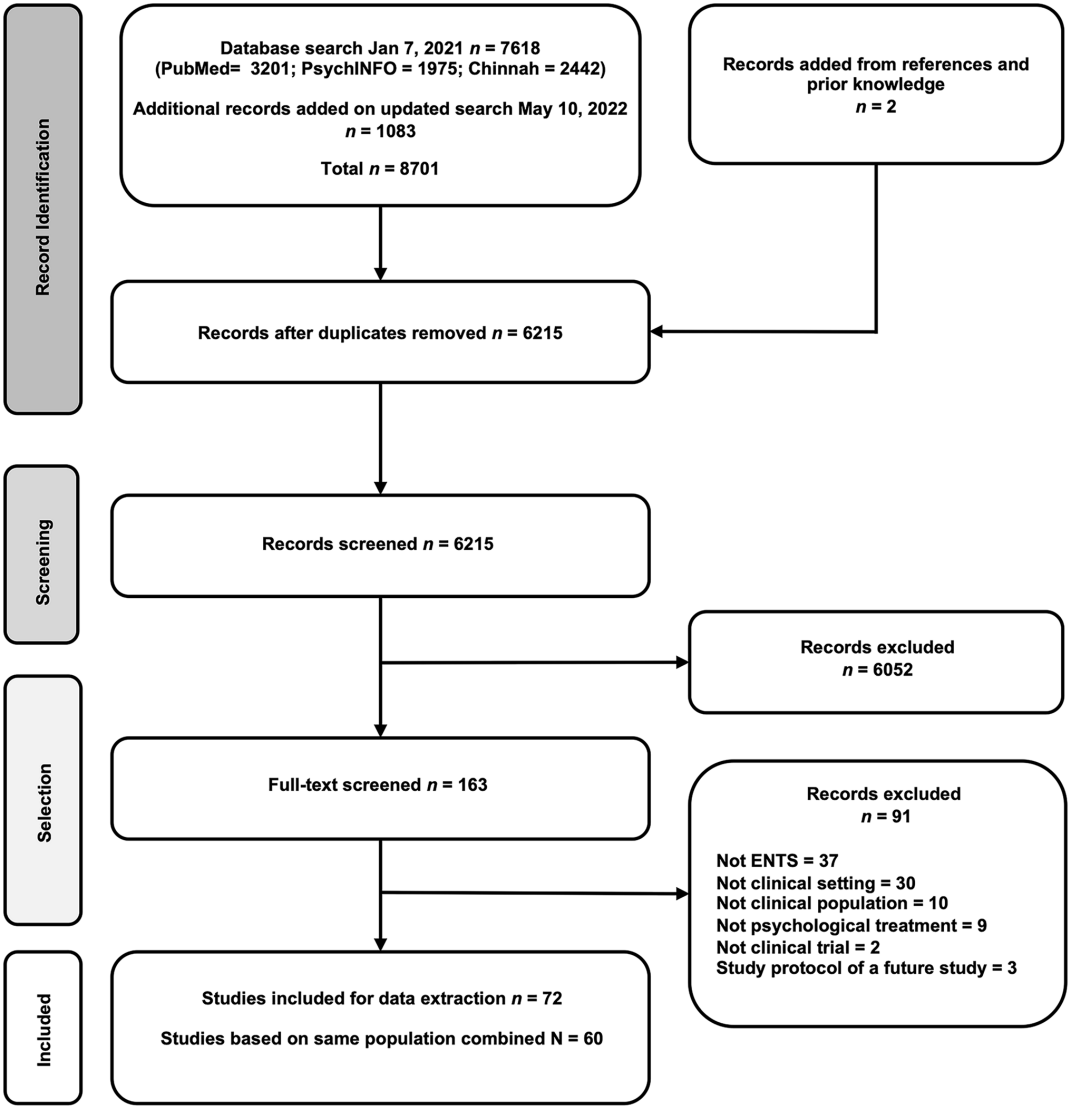
Flow chart illustrating the screening and selection process of this scoping review

The electronic search identified 8701 articles, and 6215 of these remained after duplicates were removed. Most of the titles and abstracts screened did not address clinical psychological treatments or ENTS. In total, 163 full-text articles remained after screening. Following the selection phase, 72 articles were included. However, some included papers were secondary analyses or long-term follow-ups of the same population based on the same trial. These papers were included in the data extraction process, but their information was combined in the results into one paper, using the primary report as the chief reference. This yielded a total of 60 studies, and each row in the tables is referred to as “one study” from this point onward. See Fig. 1 for a flow diagram of the screening process and reasons for exclusion.

### Study Characteristics

The included 60 studies were published between 1986 and 2022 (see Table 1). The studies primarily stem from Europe (52 studies), with the most common locations being Sweden (20 studies), followed by the Netherlands (12 studies), and England, Denmark, Finland, and Nigeria (5 studies each). In addition, three studies came from North America. The specific location of each study is described within the supplementary material.

**Table 1:**
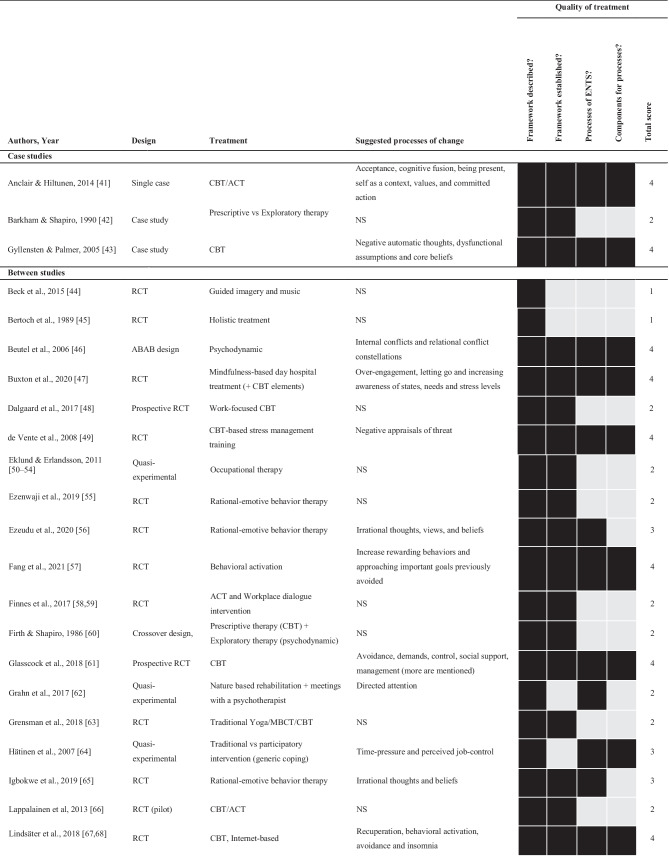

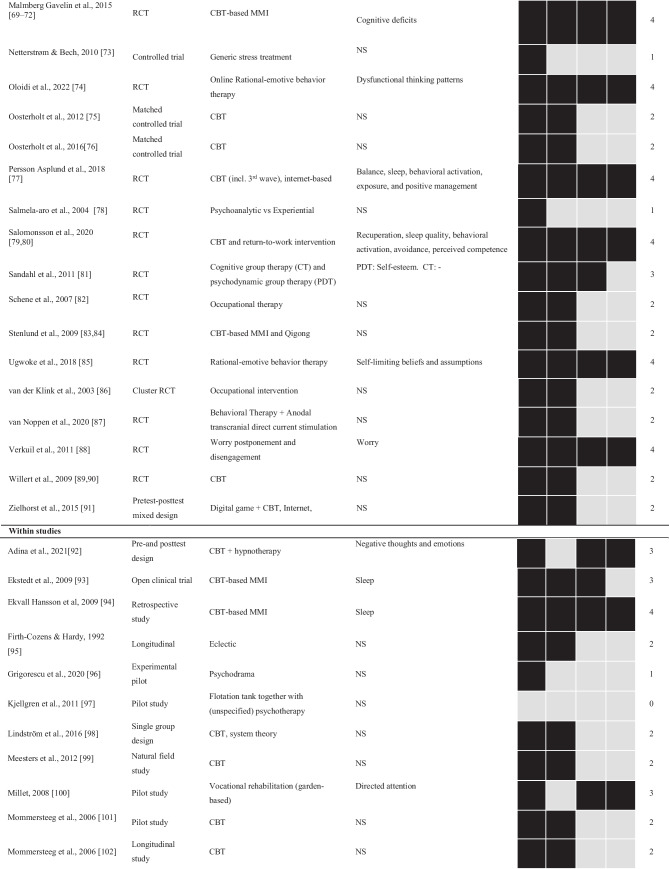

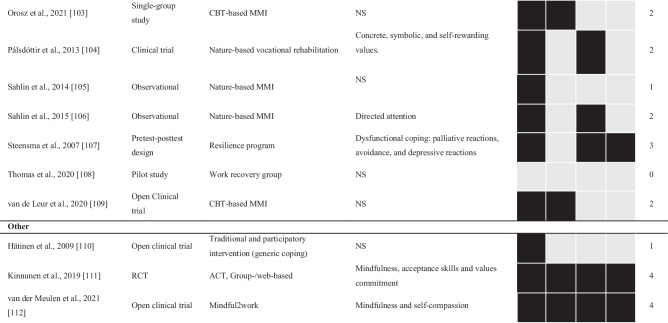
Design, treatment, suggested processes of change, and quality of treatment ratings for all of the included studies (*N* = 60)

*NS* not specified, *RCT* randomized controlled trial. Treatment: *ACT* acceptance and commitment therapy, *CBT* cognitive behavioral therapy, *MBCT* mindfulness-based cognitive therapy, *MMI* multimodal intervention

Study designs included 28 RCTs, nine single-group designs, six quasi-experimental designs, five pilot studies (of which three were with no control, one with a healthy control, and one with a no treatment control), and five open clinical trials. Furthermore, three included studies were case studies, two matched control designs, one retrospective (cohort) study, and one used a crossover design. While reporting outcomes and contents of treatment, 10% of the included studies (*n* = 6) did not specifically have the primary focus to evaluate a clinical intervention but instead other aims, such as investigating biomarkers, mediators, or sub-group trajectories during treatment [46, 101, 102, 110–112].

The average number of study participants was 89, ranging from 1 to 390, with a median of 78. The average age for participants was 42.4 (9.0), and most were women, averaging 70%. Seven included studies did not report mean age, and six did not report sex. One of the studies [66] only included male participants, while three only included women [50, 64, 105]. Detailed accounts of age and gender are provided in the supplementary material.

### Terms and Diagnoses Used for ENTS

**Fig. 2:**
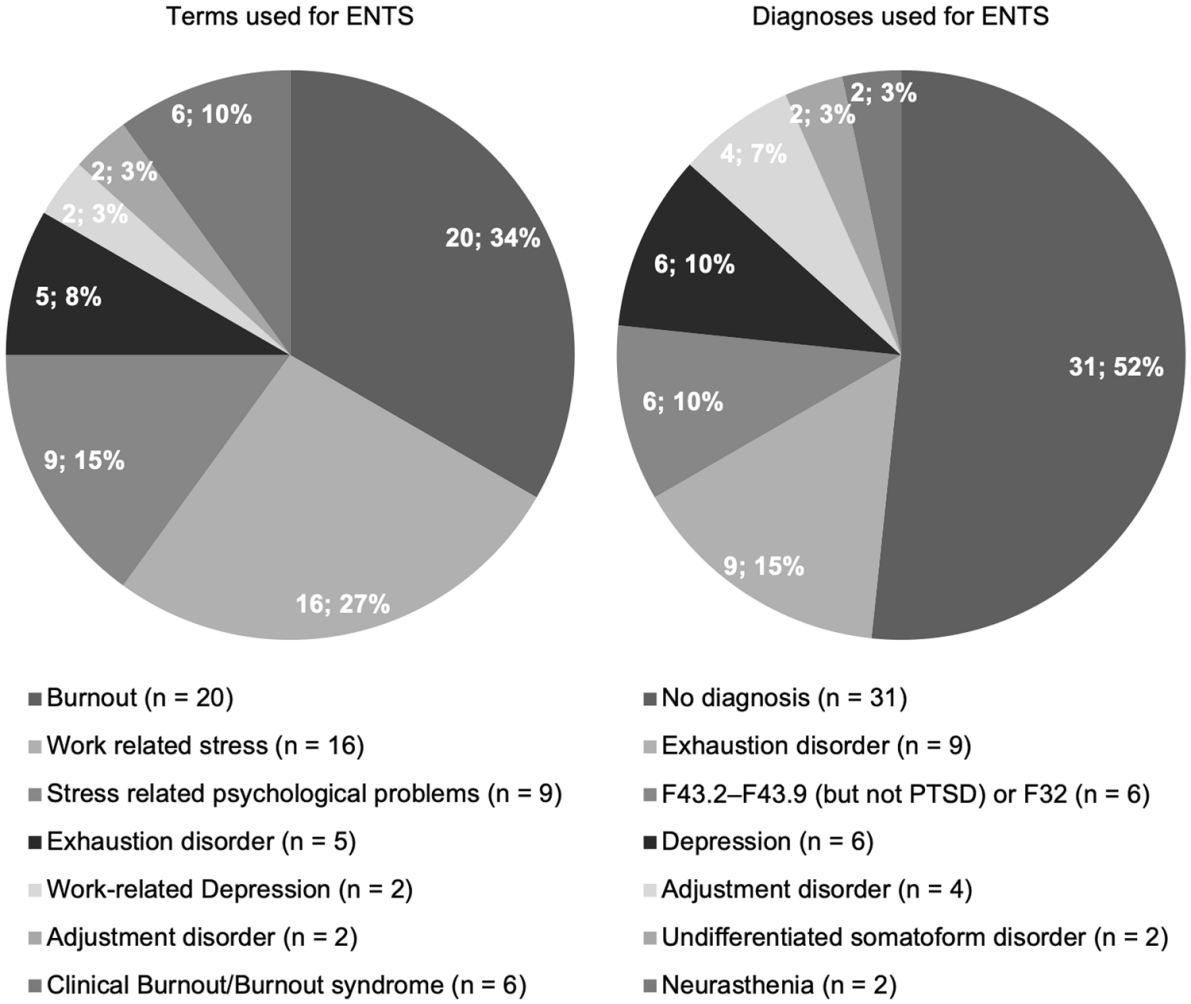
Distribution of terms and diagnoses used for exhaustion due to persistent non-traumatic stress (ENTS) in the included studies (*N* = 60)

The most common term used for ENTS was burnout (*n* = 20). In total, seven different terms were used (see Fig. 2). No diagnosis was used for ENTS in thirty-one of the studies. The most common diagnosis used was exhaustion disorder (*n* = 9). Seven different diagnoses were used in the included studies (see Fig. 2).

### Treatments Used for ENTS

Different forms of cognitive behavior therapies (CBT) were examined in 64% of the studies (*n* = 38). Of these, six studies specifically investigated rational emotive behavior therapy (REBT), five investigated CBT rehabilitation, three investigated a combination of CBT and acceptance and commitment therapy (ACT), two studies specifically studied ACT, and two used mindfulness interventions. Eclectic treatment protocols described best as generic stress-coping treatments were utilized in 10% of the included studies (*n* = 6). Furthermore, 8% of the included studies investigated psychodynamic interventions (PDT; *n* = 5), 7% investigated treatments influenced by eastern philosophy (yoga, Qigong, mindfulness; *n* = 4), and 5% occupational therapy (*n* = 3). Finally, 17% of the included studies (*n* = 10) investigated treatments deemed as “alternative,” including five focusing on nature-based rehabilitation, one on guided imagery and music, one on experiential therapy, one on psychodrama, one on flotation tank in addition to unspecified therapy, and one on hypnotherapy in addition to CBT.

Group treatment was included in 52% of the treatments (*n* = 31), and 39% of these (*n* = 12) also included individual treatment. Of the treatments, 45% (*n* = 27) were individual alone. Treatment was delivered face-to-face in 88% of the studies (*n* = 53), 10% via the Internet (*n* = 6) 2% (*n* = 1) via phone. The average treatment length was 14.7 weeks (SD = 9.4), ranging from 1 week to 1 year. Treatment length was not reported in 12% (*n* = 7) of the studies.

In 20% (*n* = 12) of the studies, no description of treatment delivery was provided. At least one psychologist or psychotherapist was involved in delivering the treatments in 50% (*n* = 30) of the studies. In 30% (*n* = 18) of the studies, treatment was delivered by professionals other than a psychologist/psychotherapist. Detailed accounts of treatment delivery, format, and length are provided in the supplementary material.

### Quality of Treatment and Processes of Change

The largest portion of the included studies, 37% (*n* = 22), received a score of two in our quality-of-treatment rating, describing a treatment framework based on well-established psychological principles (see Table 1). Of the included studies, 28% (*n* = 17) received a maximum score of 4, indicating a high-quality treatment including a treatment framework, established psychological principles, definable change processes, and components targeting these processes.

Of the 60 studies included, 48% (*n* = 29) described one or more change processes. However, seven of these described a treatment framework not based on well-established psychological principles, reducing the proportion to 37% (*n* = 22) describing change processes in conjunction with well-established psychological principles. In total, there were 16 suggested processes of change from the treatment framework with well-established psychological principles (see Box 1). Based on frequency and range across different treatments and research groups, relevant processes of change of ENTS, as suggested by published clinical trials for ENTS, are dysfunctional sleep, avoidance, behavioral activation, irrational thoughts and beliefs, perceived competence/positive management, psychological flexibility, and recuperation.
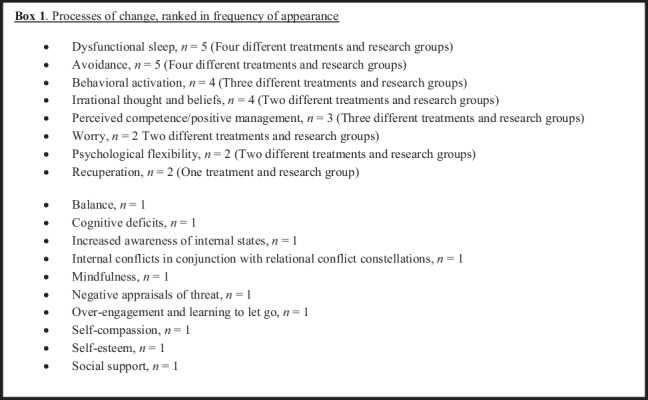


### Treatment Outcomes and Results

Detailed accounts of outcome measures and outcomes are provided in the supplementary material.

#### Measures and Use of Follow-Up

The most frequent outcome measure was sick leave/absenteeism/return to work (*n* = 16). The most employed measures of ENTS were the Shirom-Melamed Burnout Questionnaire (SMBQ; *n* = 12), the Maslach Burnout Inventory (MBI; *n* = 8), and the Oldenburg Burnout Inventory (OLBI-S; *n* = 6). For anxiety and depression, the most frequent measures used were the Beck Depression Inventory (BDI; *n* = 12), and the Hospital Anxiety and Depression Scale (HADS; *n* = 6). Short-term stress was measured most often with the Perceived Stress Scale (PSS; *n* = 10). Follow-up was assessed in 68% (*n* = 41) of the studies, and primary outcomes were explicitly defined in 27% (*n* = 16) of the studies.

#### Statistical Significance and Effect Sizes

None of the three case studies reported testing of statistical significance or effect sizes. Although, in two of the case studies [41, 43], the participants improved from pre- to post-treatment. In the third case study [42], 55–73% of the participants showed reliable clinical improvement at 6 months follow-up, depending on the outcome instrument.

Of the studies including between-group comparisons and a passive control condition, 75% (*n* = 15) reported significant outcomes (relevant for the research question and/or regarding ENTS) and 75% (*n* = 15) reported effect sizes. Effect sizes were reported in terms of Cohen’s *d* (range between 0.21 and 1.37), Hedge’s *g* (range between 1.4 and 1.8), eta-squared (range between 0.15 and 0.51), partial eta-squared (range between 0.44 and 1.0), and an unspecified effect size measure (range between 0.75 and 1.22). Of the controlled studies with at least one active control, 63% (*n* = 10) reported significant outcomes (relevant to the research question and/or regarding ENTS), and 44% (*n* = 7) reported effect sizes. Effect sizes were reported in terms of Cohen’s *d* (range between 0.26 and 1.11), Hedge’s *g* (range between 0.85 and 1.49), eta-squared (used once, *η*^2^ = 0.13), and partial eta-squared (used once, *η*^2^*p* = 0.21).

Of the studies including only within-group comparisons, 78% (*n* = 14) reported significant outcomes and 22% (*n* = 4) reported effect sizes. Effect sizes describing within-group effects were reported in terms of Cohen’s *d* (range between 0.3 and 1.8), Pearson’s *r* (range between 0.53 and 0.65), an unspecified effect size (range between 0.30 and 1.29), and percentage difference in proportions below/above an SMBQ cutoff of 4.4 (range between 23.5 and 40%).

#### High-Quality Studies

Of the 17 studies with a total score of 4 points in the treatment quality rating, 59% (*n* = 10) reported significant outcomes. Regarding treatment type, CBT was used in 30% (*n* = 3), REBT in 20% (*n* = 2), CBT/ACT in 10% (*n* = 1), mindfulness in 20% (*n* = 2), CBT-based rehabilitation in 10% (*n* = 1), and CBT and/or a return-to-work intervention in 10% (*n* = 1) of the studies. Of the studies with both a score of 4 points in the quality rating and significant outcomes, 100% (*n* = 10) were CBT- or mindfulness-based.

#### Secondary Analysis and Findings

Secondary analyses were included (within the study or as a complementary paper) in 57% (*n* = 34) of the studies. Secondary findings and results beyond treatment outcomes are described in more detail within the supplementary table. Of all secondary findings, only one was replicated across two studies, showing that the effect of Internet-CBT on symptoms of stress and exhaustion was mediated by insomnia severity [68, 80].

## Discussion

This scoping review aimed to examine and map the different definitions, diagnoses, treatments, outcome measures, and outcomes in clinical psychological treatment studies of ENTS. A further aim was to assess the quality of treatments and examine what processes of change are described within interventions for ENTS. Of the 60 studies included, 53 were based in Europe, with the most common locations being Sweden and the Netherlands. The term most associated with ENTS was burnout, and the most frequent diagnosis was exhaustion disorder. A wide array of treatments was applied, most commonly variations of CBT (68%). Psychologists or psychotherapists delivered treatment in only half of the studies, yielding some uncertainty about treatment competency and fidelity. Statistically significant results for outcomes relevant to ENTS were reported in 65% (*n* = 39) of the studies, with effect sizes ranging between 0.13 and 1.80. The most used outcome measures were sick leave/absenteeism, SMBQ (measuring burnout), and BDI (measuring depression).

Most of the included studies (73%) described a treatment framework established on well-defined psychological principles. Still, only 28% of the treatments were rated as high quality, including an established treatment framework with specific processes of change and methods targeting those processes. The most frequent change processes described were dysfunctional sleep, avoidance, behavioral activation, irrational thoughts and beliefs, worry, perceived competence/positive management, psychological flexibility, and recuperation. In summary, several different treatments, especially forms of CBT, show some promising results, but currently, there do not seem to be any uniformly established treatments, consistently adopted theoretical models or precisely specified processes of change for ENTS.

### Clinical Utility of ENTS Diagnostics Across Varying Geographical Discourses

Besides operating as a taxonomy to coordinate clinical practice and academic research, diagnoses of mental disorders should, according to the DSM-5, have clinical utility*.* More specifically, diagnoses should “help clinicians to determine prognosis, treatment plans, and potential treatment outcomes for their patients” [23]. In the current review, diagnoses were typically not used in the included studies. When used, a wide array of stress- and depression-related diagnoses is utilized (most often in the F43-category or F32-category within the ICD). Even when diagnoses are used, however, they appear to play little role in guiding treatment. Instead, it appears to be the other way around: Researchers are interested in a specific treatment for ENTS and use the available diagnoses to describe the population. Thus, the clinical utility of the diagnoses currently used for ENTS remains to be determined.

Several of the studies from Sweden use the diagnosis of exhaustion disorder, which is only applied in Sweden. Of course, diagnoses facilitate access to health care and social insurance. Perhaps not surprisingly, more than half of the included studies in the current review stem from Sweden (*n* = 20) and the Netherlands (*n* = 12), countries with extensive public healthcare and social insurance systems. The fear of increasing healthcare and social insurance costs and medicalizing “regular” work stress by accepting ENTS as a medical diagnosis is evident in the national writings discussing the matter [10, 113]. Conversely, the lack of studies originating from the USA is striking, given that the concept of burnout, to a large extent, stems from research in the USA and is widely accepted within organizational research. Perhaps the lack of clinical treatment studies from the USA reflects the absence of clinical diagnoses in the specific healthcare context of the USA.

Skepticism around the clinical utility of syndromal diagnoses is not unique to ENTS. Mental health professionals and researchers have long debated the most effective ways to define and classify mental disorders [114]. Compared to many other medical diagnoses based on biological underpinnings and signs, psychiatric diagnoses primarily rely on self-reported symptoms. Conveniently, syndromal diagnoses are clinical judgments that lack independent validation, and so what is or is not a disease is, to a large extent, socially negotiated through scientific discussion and clinical practice [115], which currently centers around the ICD and DSM.

The DSM and ICD systems assume that diagnoses reflect underlying disease processes, but there is growing agreement that the current diagnostic categories for mental disorders lack clinical utility [116]. In fact, the DSM-5 workgroup themselves have suggested that the reification of DSM-IV entities as equivalent to diseases can obscure rather than elucidate research findings [117]. So, while the validity and reliability of ENTS are faced with challenges, these challenges are shared across several mental disorders within the DSM and ICD systems. Instead of viewing ENTS as a static diagnostic category, conceptualizing it as a dynamic network of multilevel biopsychosocial processes of change may offer a promising way forward for improving international consensus on clinical interventions and theories for ENTS. Of course, this discussion is not to equate ENTS with a mental disorder but simply to note the similar challenges faced in these separate health contexts.

### Quality of Treatment and Treatment Theory for ENTS

In our quality ratings, the largest proportion of the included studies, 37% (*n* = 22), received a score of two describing a treatment framework based on well-established psychological principles. Today CBT is a broad family of methods with many alternate or even subtly conflicting theories and underlying assumptions. Therefore, only describing treatment as CBT leaves considerable latitude regarding the treatment’s theory, focus, and contents.

While 37% (*n* = 22) of the included papers described some form of the change process in conjunction with well-established psychological principles, few explicitly define how these specific change processes relate to the origin and maintenance of ENTS. The lack of theory-driven evidence-based treatments for stress-related conditions such as ENTS, compared to anxiety and mood disorders, has previously been highlighted [79]. One possible explanation could be the monocausal latent disease model inherent in a syndromal approach, favoring biologic reductionism over a complex multilevel view [118]. From a biomechanical view, persistent stress symptoms are regarded as the result of a physiological resource-demand imbalance rather than a consequence of interacting physiological, behavioral, cognitive, emotional, and social elements [119]. Such biomechanical perspectives risk fostering generic methods focusing on problem-solving, relaxation, and coping rather than encouraging the development of treatment theory about what specific processes initiate and maintain ENTS. Consequently, future clinical treatment models would probably benefit from focusing more on such processes to promote the development of theory-driven, evidence-based treatments for ENTS. This includes exploring what progressive step-by-step changes in physiological, behavioral, cognitive, emotional, and social domains result in severe debilitating exhaustion symptoms in the context of persistent non-traumatic stress.

### Processes of Change — Toward a New Treatment Perspective on ENTS

In recent years, a group of prominent researchers in clinical psychology have set out to change the agenda for modern clinical interventional research: away from syndrome-based diagnoses and syndrome-specific protocol-based interventions and toward a process-based approach to treatment [33]. Instead of a monocausal latent disease model for understanding illnesses, a network approach is recommended, conceptualizing mental illness from a multicausal network of interacting symptoms and associated processes, where specific syndromes are the result of dynamically self-reinforcing clusters [120, 121]. Process-based therapy adopts an idiographic perspective where treatment models should have explicit philosophical assumptions, be theoretically coherent and enable clinicians to select components to target established, evidence-based, and modifiable change processes tailored to specific individuals [122].

In a recent literature review on the mediation of psychosocial interventions for mental health, Hayes et al. [123] identified 66 replicated measures of unique mediators, accounting for 281 specific findings across 204 studies [123]. Of the most frequent processes of change identified in the current review, behavioral activation, worry, and psychological flexibility are all represented. While not described in the review of Hayes et al. [123], avoidance and perceived competence are incorporated within several overlapping constructs, such as self-efficacy, non-reactivity to inner experience, and perceived control in the present. Furthermore, approaching behaviors in line with values, rather than being governed by negatively reinforced avoidance, constitute a central part of psychological flexibility [124].

Given the complexity and potential heterogeneity of ENTS, together with the current lack of consensus in international nomenclatural, it seems advisable to adopt an approach to treatment delivery that can customize itself around individual participants’ needs rather than assuming one set protocol will best fit everyone. Furthermore, a process-based approach could shift the academic focus of ENTS over time. This focus could shift away from whether ENTS exists “by its own right” as a latent disease within the already debated diagnostic systems, where low specificity and high comorbidity are the norm rather than the rule [116]. Instead, the focus could shift toward an understanding of what change processes generate and maintain ENTS for particular people in specific contexts. Such an approach could potentially enable research that results in better psychological interventions with higher clinical utility and, consequently, better treatment outcomes for individuals with ENTS. One first step toward such an approach would be for future research to focus on optimizing effects on established change processes currently utilized within ENTS treatment.

### Limitations

One limitation of the current review is it is a scoping review and not a systematic review of efficacy — this remains to be done. Since the specific aim of this review was mapping the literature, the large number of trials is summarized into frequency counts or percentages at the risk of concealing individual yet essential clinical findings. Many of the included studies contained potentially important secondary findings, such as trajectories for patients going through long-term rehabilitation for burnout [110] or economic outcomes. For example, it appears that using ACT, work-directed intervention (WDI), and a combination of both reduced healthcare costs, whereas WDI alone reduced welfare costs [59]. Other interesting results relate to differences in lower cortisol levels at awakening [101, 102] or reductions in the arousal from sleep being predictive of decreased exhaustion symptoms during treatment [93]. While described within the supplementary table, these were outside the scope of the current review. A different approach and review methods focusing beyond simply “scoping” could reveal nuances and findings not made available with the current descriptive approach.

One significant limitation of the current review is that its findings are primarily based on European studies. This geographic discrepancy may be due to the focus of the intervention on clinical treatments for stress-related disorders, which are seldom covered by public healthcare and social insurance systems. Additionally, differences in cultural conceptualizations of ENTS and what is considered a condition warranting professional healthcare interventions could contribute to these disparities. To enhance the understanding of the definitions, treatments, and theories surrounding ENTS, it may be beneficial to expand the scope beyond clinical interventions published in English scientific journals. This could involve exploring non-Western sources and incorporating perspectives from diverse cultural and linguistic backgrounds. Such an approach could provide valuable insights into the cross-cultural variations in the manifestation and treatment of ENTS.

In the clinical research literature, the terms “mechanisms of change,” “mediators,” and “processes of change” are often used interchangeably with different underlying definitions and assumptions. This is understandable, given the relative novelty of these concepts in the field. In this review, an inclusive approach was used to capture all potential change processes available and to avoid judging previous research efforts based on the new standards of process-based therapy. A stricter definition of change processes in the current review focusing on the quality of the description rather than frequency across studies may have led to more limited but more informative results. Going forward, a coherent definition of change processes is encouraged in developing ENTS treatments since this would make translating findings across different treatments and studies easier.

Lastly, while our quality assessment is based on clinical recommendations, it is not an established rating system and has not been utilized elsewhere. What is to be considered a high-quality psychological treatment is a question not easily answered and will vary across different perspectives within clinical psychology. The current scoping review has strived for transparency regarding its assumptions about what a psychological treatment of high quality is, but different assumptions would most certainly have yielded different quality assessment results.

## Conclusion

Several psychological treatments for ENTS, primarily forms of CBT, show promising results. Currently, however, there seem to be no well-established treatments, theoretical models, or processes of change established for ENTS. Instead of utilizing a monocausal, syndromal, and potentially bio-reductionist perspective on ENTS, future clinical research efforts would probably benefit from embracing a process-based approach to treatment, as presently reflected in the wider field of clinical psychology. An appropriate place to start may be those already established change processes identified in the current review: avoidance, psychological flexibility, self-efficacy, worry, and irrational thoughts and beliefs. Perhaps focusing on multilevel biopsychosocial change processes could help bridge the gap between the current lack of theory-driven, evidence-based treatments for ENTS compared to anxiety and mood disorders.

### Supplementary Information

Below is the link to the electronic supplementary material.

Supplementary file1 (PDF 824 KB)

## References

[1] Anderson NB. Levels of Analysis in health science: a framework for integrating sociobehavioral and biomedical research. Ann N Y Acad Sci [Internet]. 1998 May;840(1):563–76. Available from: https://onlinelibrary.wiley.com/doi/10.1111/j.1749-6632.1998.tb09595.x. 10.1111/j.1749-6632.1998.tb09595.x 9629283

[2] Baum A, Posluszny DM. Health psychology: Mapping biobehavioral contributions to health and illness. Annu Rev Psychol [Internet]. 1999 Feb;50(1):137–63. Available from: https://www.annualreviews.org/doi/10.1146/annurev.psych.50.1.137. 10.1146/annurev.psych.50.1.137 10074676

[3] Cohen S, Janicki-Deverts D, Miller GE. Psychological stress and disease. JAMA [Internet]. 2007 Oct 10;298(14):1685–7. Available from: 10.1001/jama.298.14.1685. 10.1001/jama.298.14.1685 17925521

[4] Grossi G, Perski A, Osika W, Savic I. Stress‐related exhaustion disorder – clinical manifestation of burnout? A review of assessment methods, sleep impairments, cognitive disturbances, and neuro‐biological and physiological changes in clinical burnout. Scand J Psychol [Internet]. 2015 Dec 23;56(6):626–36. Available from: https://onlinelibrary.wiley.com/doi/10.1111/sjop.12251. 10.1111/sjop.12251 26496458

[5] OECD. Mental Health and Work: Sweden [Internet]. OECD; 2013. 134 p. (Mental Health and Work). Available from: https://www.oecd-ilibrary.org/employment/mental-health-and-work-sweden_9789264188730-en.

[6] OECD. Sick on the Job? [Internet]. OECD; 2012. 212 p. (Mental Health and Work). Available from: https://www.oecd-ilibrary.org/social-issues-migration-health/mental-health-and-work_9789264124523-en.

[7] Hewlett E, Moran V. Making mental health count: the social and economic costs of neglecting mental health care [Internet]. OECD Health Policy Studies. OECD; 2014 [cited 2019 Jun 17]. 1–8 p. (Mental Health and Work). Available from: https://www.oecd-ilibrary.org/social-issues-migration-health/mental-health-and-work_9789264124523-en.

[8] European Agency for Safety and Health at Work, Hassard J, Teoh K, Cox T, Dewe P, Cosmar M, et al. Calculating the cost of work-related stress and psychosocial risks: European risk observatory: literature review. www.Healthy-Workplaces.Eu. 2014. 1–42 p.

[9] Eurofound. Burnout in the workplace: a review of data and policy responses in the EU [Internet]. Publications Office of the European Union. Luxemburg: Publications Office of the European Union; 2018. 1–41 p. Available from: http://eurofound.link/ef18047.

[10] Cléach O. Claude Veil, Vulnérabilités au travail. Naissance et actualité de la psychopathologie du travail. Recueil de textes présentés par Dominique Lhuilier, Toulouse, Erès, 2012. La Nouvelle Revue du Travail [Internet]. 2014 May 1;(4). Available from: http://journals.openedition.org/nrt/1765.

[11] van der Klink JJ, van Dijk FJ. Dutch practice guidelines for managing adjustment disorders in occupational and primary health care. Scand J Work Environ Health [Internet]. 2003 Dec;29(6):478–87. Available from: http://www.sjweh.fi/show_abstract.php?abstract_id=756. 10.5271/sjweh.756 14712856

[12] Jensen JH, Miskowiak KW, Purdon SE, Thomsen JF, Eller NH. Screening for cognitive impairment among patients with work-related stress complaints in Denmark: validation and evaluation of objective and self-report tools. Scand J Work Environ Health [Internet]. 2022 Jan 1;48(1):71–80. Available from: http://www.sjweh.fi/show_abstract.php?abstract_id=3990. 10.5271/sjweh.3990 PMC8729168 34580738

[13] Engebretsen KM, Bjorbækmo WS. Naked in the eyes of the public: a phenomenological study of the lived experience of suffering from burnout while waiting for recognition to be ill. J Eval Clin Pract [Internet]. 2019 Dec 25;25(6):1017–26. Available from: https://onlinelibrary.wiley.com/doi/10.1111/jep.13244. 10.1111/jep.13244 31342598

[14] Tylec A, Kot E, Kostecka B, Kiełczewska C, Kania A, Siejko K, et al. Burnout syndrome and empathy and altruism level among nurses - the preliminary study. Kwartalnik Naukowy Fides et Ratio [Internet]. 2021 Sep 30;47(3):462–75. Available from: https://fidesetratio.com.pl/ojs/index.php/FetR/article/view/923.

[15] Wallensten J, Åsberg M, Wiklander M, Nager A. Role of rehabilitation in chronic stress-induced exhaustion disorder: a narrative review. J Rehabil Med [Internet]. 2019;51(5):331–42. Available from: https://www.medicaljournals.se/jrm/content/abstract/10.2340/16501977-2545. 10.2340/16501977-2545 30882887

[16] Lindsäter E, Svärdman F, Wallert J, Ivanova E, Söderholm A, Fondberg R, et al. Exhaustion disorder: scoping review of research on a recently introduced stress-related diagnosis. BJ Psych Open [Internet]. 2022 Sep 24;8(5):e159. Available from: https://www.cambridge.org/core/product/identifier/S2056472422005592/type/journal_article. 10.1192/bjo.2022.559 PMC9438479 36458830

[17] Beard G. Neurasthenia, or nervous exhaustion. Boston Med Surg J [Internet]. 1869 Apr 29;80(13):217–21. Available from: http://www.nejm.org/doi/abs/10.1056/NEJM186904290801301.

[18] Kraepelin E, Diefendorf AR. Clinical psychiatry: a text-book for students and physicians; abstracted and adapted from the sixth German edition of Kraepelin’s Lehrbuch Der Psychiatrie. Franklin Classics; 2018.

[19] Taylor RE. Death of neurasthenia and its psychological reincarnation. British J Psychiat [Internet]. 2001 Dec 2;179(6):550–7. Available from: https://www.cambridge.org/core/product/identifier/S0007125000160131/type/journal_article. 10.1192/bjp.179.6.550 11731361

[20] Neckel S, Schaffner AK, Wagner G. Burnout, fatigue, exhaustion [Internet]. Neckel S, Schaffner AK, Wagner G, editors. Burnout, fatigue, exhaustion: an interdisciplinary perspective on a modern affliction. Cham: Springer International Publishing; 2017. 1–316 p. Available from: http://link.springer.com/10.1007/978-3-319-52887-8.

[21] Schaufeli WB, Leiter MP, Maslach C. Burnout: 35 years of research and practice. Car Dev Intern [Internet]. 2009 Jun 19;14(3):204–20. Available from: https://www.emerald.com/insight/content/doi/10.1108/13620430910966406/full/html.

[22] World Health Organization (1992). The ICD-10 classification of mental and behavioural disorders.

[23] American Psychiatric Association (2013). Diagnostic and statistical manual of mental disorders : DSM-5.

[24] van Dam A. A clinical perspective on burnout: diagnosis, classification, and treatment of clinical burnout. Euro J Work Organ Psychol [Internet]. 2021 Sep 3;30(5):732–41. Available from: https://www.tandfonline.com/doi/full/10.1080/1359432X.2021.1948400.

[25] Maslach C, Leiter MP. Understanding the burnout experience: recent research and its implications for psychiatry. World Psychiatry [Internet]. 2016 Jun 1;15(2):103–11. Available from: https://onlinelibrary.wiley.com/doi/10.1002/wps.20311. 10.1002/wps.20311 PMC4911781 27265691

[26] Bianchi R, Schonfeld IS, Laurent E. Burnout-depression overlap: a review. Clin Psychol Rev [Internet]. 2015;36:28–41. Available from: 10.1016/j.cpr.2015.01.004. 10.1016/j.cpr.2015.01.004 25638755

[27] Bianchi R, Schonfeld IS, Laurent E. Burnout: moving beyond the status quo. Int J Stress Manag [Internet]. 2019 Feb;26(1):36–45. Available from: http://doi.apa.org/getdoi.cfm?doi=10.1037/str0000088.

[28] Schonfeld IS, Bianchi R. Burnout and depression: two entities or one? J Clin Psychol [Internet]. 2016 Jan;72(1):22–37. Available from: https://onlinelibrary.wiley.com/doi/10.1002/jclp.22229. 10.1002/jclp.22229 26451877

[29] Koutsimani P, Montgomery A, Georganta K. The relationship between burnout, depression, and anxiety: a systematic review and meta-analysis. Front Psychol [Internet]. 2019 Mar 13;10(MAR). Available from: https://www.frontiersin.org/article/10.3389/fpsyg.2019.00284/full. 10.3389/fpsyg.2019.00284 PMC6424886 30918490

[30] Wallensten J, Mobarrez F, Åsberg M, Borg K, Beser A, Wilczek A, et al. Isoforms of soluble vascular endothelial growth factor in stress-related mental disorders: a cross-sectional study. Sci Rep. 2021;11(1). 10.1038/s41598-021-96313-8 PMC8370974 34404878

[31] Orosz A, Federspiel A, Haisch S, Seeher C, Dierks T, Cattapan K. A biological perspective on differences and similarities between burnout and depression. Neurosci Biobehav Rev [Internet]. 2017 Feb 1;73:112–22. Available from: https://linkinghub.elsevier.com/retrieve/pii/S0149763416301269. 10.1016/j.neubiorev.2016.12.005 27993607

[32] Nadon L, De Beer LT, Morin AJS. Should burnout be conceptualized as a mental disorder? Behavioral Sciences [Internet]. 2022 Mar 17;12(3):82. Available from: https://www.mdpi.com/2076-328X/12/3/82. 10.3390/bs12030082 PMC8945132 35323401

[33] Hayes SC, Hofmann SG, Stanton CE, Carpenter JK, Sanford BT, Curtiss JE (2019). The role of the individual in the coming era of process-based therapy. Behav Res Ther.

[34] Kazdin AE. Mediators and mechanisms of change in psychotherapy research. Annu Rev Clin Psychol [Internet]. 2007 Apr 1;3(1):1–27. Available from: https://www.annualreviews.org/doi/10.1146/annurev.clinpsy.3.022806.091432. 10.1146/annurev.clinpsy.3.022806.091432 17716046

[35] Hofmann SG, Hayes SC. The future of intervention science: process-based therapy. Clin Psychol Sci [Internet]. 2019 Jan 29;7(1):37–50. Available from: http://journals.sagepub.com/doi/10.1177/2167702618772296. 10.1177/2167702618772296 PMC6350520 30713811

[36] Munn Z, Peters MDJ, Stern C, Tufanaru C, McArthur A, Aromataris E. Systematic review or scoping review? Guidance for authors when choosing between a systematic or scoping review approach. BMC Med Res Methodol [Internet]. 2018 Dec 19;18(1):143. Available from: https://bmcmedresmethodol.biomedcentral.com/articles/10.1186/s12874-018-0611-x. 10.1186/s12874-018-0611-x PMC6245623 30453902

[37] Peters M, Godfrey C, McInerney P, Munn Z, Trico A, Khalil H. Chapter 11: scoping reviews. In: JBI Manual for Evidence Synthesis [Internet]. JBI; 2020. Available from: https://wiki.jbi.global/display/MANUAL/Chapter+11%3A+Scoping+reviews.

[38] Tricco AC, Lillie E, Zarin W, O’Brien KK, Colquhoun H, Levac D, et al. PRISMA extension for scoping reviews (PRISMA-ScR): checklist and explanation. Ann Intern Med [Internet]. 2018 Oct 2;169(7):467–73. Available from: https://www.acpjournals.org/doi/10.7326/M18-0850. 10.7326/M18-0850 30178033

[39] Higgins JPT, Thomas J, Chandler J, Cumpston M, Li T, Page MJ, et al., editors. Cochrane handbook for systematic reviews of interventions [Internet]. Wiley; 2019. Available from: https://onlinelibrary.wiley.com/doi/book/10.1002/9781119536604. 10.1002/14651858.ED000142 PMC10284251 31643080

[40] Hayes SC, Long DM, Levin ME, Follette WC. Treatment development: can we find a better way? Clin Psychol Rev [Internet]. 2013 Nov;33(7):870–82. Available from: https://linkinghub.elsevier.com/retrieve/pii/S0272735813000500. 10.1016/j.cpr.2012.09.009 23647855

[41] Anclair M, Hiltunen AJ. Cognitive behavioral therapy for stress-related problems. Clin Case Stud [Internet]. 2014 Dec 12;13(6):472–86. Available from: http://journals.sagepub.com/doi/10.1177/1534650114522090.

[42] Barkham M, Shapiro DA. Brief psychotherapeutic interventions for job-related distress: a pilot study of prescriptive and exploratory therapy. Couns Psychol Q [Internet]. 1990 Apr 27;3(2):133–47. Available from: http://www.tandfonline.com/doi/full/10.1080/09515079008254242.

[43] Gyllensten K, Palmer S. Working with a client suffering from workplace stress in a primary care setting: a cognitive behavioural case study [Internet]. 2005. Available from: https://www.researchgate.net/publication/335987906.

[44] Beck BD, Hansen ÅM, Gold C. Coping with work-related stress through guided imagery and Music (GIM): randomized controlled trial. J Music Ther [Internet]. 2015;52(3):323–52. Available from: https://academic.oup.com/jmt/article-lookup/doi/10.1093/jmt/thv011. 10.1093/jmt/thv011 26424362

[45] Bertoch MR, Nielsen EC, Curley JR, Borg WR. Reducing teacher stress. The Journal of Experimental Education [Internet]. 1989 Jan 16;57(2):117–28. Available from: http://www.tandfonline.com/doi/abs/10.1080/00220973.1989.10806500.

[46] Beutel ME, Knickenberg RJ, Krug B, Mund S, Schattenburg L, Zwerenz R. Psychodynamic focal group treatment for psychosomatic inpatients–with an emphasis on work-related conflicts. Int J Group Psychother [Internet]. 2006 Jul 21;56(3):285–306. Available from: https://www.tandfonline.com/doi/full/10.1521/ijgp.2006.56.3.285. 10.1521/ijgp.2006.56.3.285 16822177

[47] Buxton AE, Remmers C, Unger HP, Plinz N, Michalak J. Treating depression mindfully in a day hospital: a randomised controlled pilot study. Mindfulness (N Y) [Internet]. 2020 Feb 28;11(2):384–400. Available from: http://link.springer.com/10.1007/s12671-019-01233-4.

[48] Dalgaard VL, Andersen LPS, Andersen JH, Willert MV, Carstensen O, Glasscock DJ. Work-focused cognitive behavioral intervention for psychological complaints in patients on sick leave due to work-related stress: results from a randomized controlled trial. J Negat Results Biomed [Internet]. 2017 Dec 22;16(1):13. Available from: http://jnrbm.biomedcentral.com/articles/10.1186/s12952-017-0078-z. 10.1186/s12952-017-0078-z PMC5567478 28830555

[49] de Vente W, Kamphuis JH, Emmelkamp PMG, Blonk RWB. Individual and group cognitive-behavioral treatment for work-related stress complaints and sickness absence: a randomized controlled trial. J Occup Health Psychol [Internet]. 2008 Jul;13(3):214–31. Available from: http://doi.apa.org/getdoi.cfm?doi=10.1037/1076-8998.13.3.214. 10.1037/1076-8998.13.3.214 18572993

[50] Eklund M, Erlandsson LK. Return to work outcomes of the redesigning daily occupations (ReDO) program for women with stress-related disorders—a comparative study. Women Health [Internet]. 2011 Nov;51(7):676–92. Available from: http://www.tandfonline.com/doi/abs/10.1080/03630242.2011.618215. 10.1080/03630242.2011.618215 22082247

[51] Eklund M, Erlandsson LK. Quality of life and client satisfaction as outcomes of the redesigning daily occupations (ReDO) programme for women with stress-related disorders: a comparative study. Work [Internet]. 2013 Sep 27;46(1):51–8. Available from: https://www.medra.org/servlet/aliasResolver?alias=iospress&doi=10.3233/WOR-121524. 10.3233/WOR-121524 23324689

[52] Wästberg BA, Erlandsson LK, Eklund M. Women’s perceived work environment after stress-related rehabilitation: experiences from the ReDO project. Disabil Rehabil [Internet]. 2016 Mar 12;38(6):528–34. Available from: http://www.tandfonline.com/doi/full/10.3109/09638288.2015.1046567. 10.3109/09638288.2015.1046567 26171915

[53] Eklund M. Anxiety, depression, and stress among women in work rehabilitation for stress-related disorders. Int J Ment Health [Internet]. 2013 Dec 10;42(4):34–47. Available from: https://www.tandfonline.com/doi/full/10.2753/IMH0020-7411420402.

[54] Eklund M. Minor long-term effects 3–4 years after the ReDO^TM^ intervention for women with stress-related disorders: a focus on sick leave rate, everyday occupations and well-being. Work [Internet]. 2017 Dec 13;58(4):527–36. Available from: https://www.medra.org/servlet/aliasResolver?alias=iospress&doi=10.3233/WOR-172639. 10.3233/WOR-172639 29254124

[55] Ezenwaji IO, Eseadi C, Ugwoke SC, Vita-Agundu UC, Edikpa E, Okeke FC, et al. A group-focused rational emotive behavior coaching for management of academic burnout among undergraduate students. Medicine [Internet]. 2019 Jul 1;98(30):e16352. Available from: https://journals.lww.com/00005792-201907260-00010. 10.1097/MD.0000000000016352 PMC6708802 31348235

[56] Ezeudu FO, Nwoji IHN, Dave-Ugwu PO, Abaeme DO, Ikegbunna NR, Agugu CV, et al. Intervention for burnout among chemistry education undergraduates in Nigeria. J Intern Med Res [Internet]. 2020 Jan 9;48(1):030006051986783. Available from: http://journals.sagepub.com/doi/10.1177/0300060519867832. 10.1177/0300060519867832 PMC7262822 31394945

[57] Fang CM, McMahon K, Miller ML, Rosenthal MZ. A pilot study investigating the efficacy of brief, phone‐based, behavioral interventions for burnout in graduate students. J Clin Psychol [Internet]. 2021 Dec 13;77(12):2725–45. Available from: https://onlinelibrary.wiley.com/doi/10.1002/jclp.23245. 10.1002/jclp.23245 PMC8688279 34517431

[58] Finnes A, Enebrink P, Sampaio F, Sorjonen K, Dahl J, Ghaderi A, et al. Cost-effectiveness of acceptance and commitment therapy and a workplace intervention for employees on sickness absence due to mental disorders. J Occup Environ Med [Internet]. 2017;59(12):1211–20. Available from: http://www.ncbi.nlm.nih.gov/pubmed/28953070. 10.1097/JOM.0000000000001156 28953070

[59] Finnes A, Hoch JS, Enebrink P, Dahl J, Ghaderi A, Nager A, et al. Economic evaluation of return-to-work interventions for mental disorder-related sickness absence: two years follow-up of a randomized clinical trial. Scand J Work Environ Health [Internet]. 2022 May 1;48(4):264–72. Available from: http://www.sjweh.fi/show_abstract.php?abstract_id=4012. 10.5271/sjweh.4012 PMC9524165 35094095

[60] Firth J, Shapiro DA. An evaluation of psychotherapy for job-related distress. J Occup Psychol [Internet]. 1986 Jun;59(2):111–9. Available from: https://onlinelibrary.wiley.com/doi/10.1111/j.2044-8325.1986.tb00218.x.

[61] Glasscock DJ, Carstensen O, Dalgaard VL. Recovery from work-related stress: a randomized controlled trial of a stress management intervention in a clinical sample. Int Arch Occup Environ Health [Internet]. 2018 Aug 28;91(6):675–87. Available from: http://link.springer.com/10.1007/s00420-018-1314-7. 10.1007/s00420-018-1314-7 29808433

[62] Grahn P, Pálsdóttir AM, Ottosson J, Jonsdottir IH. Longer nature-based rehabilitation may contribute to a faster return to work in patients with reactions to severe stress and/or depression. Int J Environ Res Public Health [Internet]. 2017 Oct 27;14(11):1310. Available from: http://www.mdpi.com/1660-4601/14/11/1310. 10.3390/ijerph14111310 PMC5707949 29076997

[63] Grensman A, Acharya BD, Wändell P, Nilsson GH, Falkenberg T, Sundin Ö, et al. Effect of traditional yoga, mindfulness–based cognitive therapy, and cognitive behavioral therapy, on health related quality of life: a randomized controlled trial on patients on sick leave because of burnout. BMC Complement Altern Med [Internet]. 2018 Dec 6;18(1):80. Available from: https://bmccomplementalternmed.biomedcentral.com/articles/10.1186/s12906-018-2141-9. 10.1186/s12906-018-2141-9 PMC5839058 29510704

[64] Hätinen M, Kinnunen U, Pekkonen M, Kalimo R. Comparing two burnout interventions: perceived job control mediates decreases in burnout. Int J Stress Manag [Internet]. 2007 Aug;14(3):227–48. Available from: http://doi.apa.org/getdoi.cfm?doi=10.1037/1072-5245.14.3.227.

[65] Igbokwe UL, Nwokenna EN, Eseadi C, Ogbonna CS, Nnadi EM, Ololo KO, et al. Intervention for burnout among English education undergraduates: implications for curriculum innovation. Medicine [Internet]. 2019 Jun 1;98(26):e16219. Available from: https://journals.lww.com/00005792-201906280-00088. 10.1097/MD.0000000000016219 PMC6617034 31261577

[66] Lappalainen P, Kaipainen K, Lappalainen R, Hoffrén H, Myllymäki T, Kinnunen ML, et al. Feasibility of a personal health technology-based psychological intervention for men with stress and mood problems: randomized controlled pilot trial. JMIR Res Protoc [Internet]. 2013 Jan 9;2(1):e1. Available from: http://www.researchprotocols.org/2013/1/e1/. 10.2196/resprot.2389 PMC3628150 23611946

[67] Lindsäter E, Axelsson E, Salomonsson S, Santoft F, Ejeby K, Ljótsson B, et al. Internet-based cognitive behavioral therapy for chronic stress: a randomized controlled trial. Psychother Psychosom [Internet]. 2018;87(5):296–305. Available from: https://www.karger.com/Article/FullText/490742. 10.1159/000490742 30041167

[68] Lindsäter E, Axelsson E, Salomonsson S, Santoft F, Ljótsson B, Åkerstedt T, et al. The mediating role of insomnia severity in internet-based cognitive behavioral therapy for chronic stress: secondary analysis of a randomized controlled trial. Behav Res Ther [Internet]. 2021 Jan;136(April 2020):103782. Available from: https://linkinghub.elsevier.com/retrieve/pii/S0005796720302369. 10.1016/j.brat.2020.103782 33276274

[69] Gavelin HM, Boraxbekk CJ, Stenlund T, Järvholm LS, Neely AS. Effects of a process-based cognitive training intervention for patients with stress-related exhaustion. Stress [Internet]. 2015 Sep 3;18(5):578–88. Available from: http://www.tandfonline.com/doi/full/10.3109/10253890.2015.1064892. 10.3109/10253890.2015.1064892 26305186

[70] Eskilsson T, Slunga Järvholm L, Malmberg Gavelin H, Stigsdotter Neely A, Boraxbekk CJ. Aerobic training for improved memory in patients with stress-related exhaustion: a randomized controlled trial. BMC Psychiatry [Internet]. 2017 Dec 2;17(1):322. Available from: https://bmcpsychiatry.biomedcentral.com/articles/10.1186/s12888-017-1457-1. 10.1186/s12888-017-1457-1 PMC5581420 28865430

[71] Gavelin HM, Neely AS, Andersson M, Eskilsson T, Järvholm LS, Boraxbekk CJ. Neural activation in stress-related exhaustion: cross-sectional observations and interventional effects. Psychiatry Res Neuroimaging [Internet]. 2017 Nov;269:17–25. Available from: https://linkinghub.elsevier.com/retrieve/pii/S0925492717300549. 10.1016/j.pscychresns.2017.08.008 28917154

[72] Malmberg Gavelin H, Eskilsson T, Boraxbekk CJ, Josefsson M, Stigsdotter Neely A, Slunga Järvholm L. Rehabilitation for improved cognition in patients with stress-related exhaustion disorder: RECO – a randomized clinical trial. Stress [Internet]. 2018 Jul 4;21(4):279–91. Available from: https://www.tandfonline.com/doi/full/10.1080/10253890.2018.1461833. 10.1080/10253890.2018.1461833 29693483

[73] Netterstrøm B, Bech P. Effect of a multidisciplinary stress treatment programme on the return to work rate for persons with work-related stress. A non-randomized controlled study from a stress clinic. BMC Public Health [Internet]. 2010 Dec 1;10(1):658. Available from: https://bmcpublichealth.biomedcentral.com/articles/10.1186/1471-2458-10-658. 10.1186/1471-2458-10-658 PMC3091572 21040559

[74] Oloidi FJ, Sewagegn AA, Amanambu OV, Umeano BC, Ilechukwu LC, Palmieri F. Academic burnout among undergraduate history students: effect of an intervention. Medicine [Internet]. 2022;101(7):e28886–e28886. Available from: https://search.ebscohost.com/login.aspx?direct=true&db=cin20&AN=155473309&site=ehost-live. 10.1097/MD.0000000000028886 PMC9282037 35363205

[75] Oosterholt BG, van der Linden D, Maes JH, Verbraak MJ, Kompier MA. Burned out cognition – cognitive functioning of burnout patients before and after a period with psychological treatment. Scand J Work Environ Health [Internet]. 2012 Jul;38(4):358–69. Available from: http://www.sjweh.fi/show_abstract.php?abstract_id=3256. 10.5271/sjweh.3256 22025205

[76] Oosterholt BG, Maes JHR, van der Linden D, Verbraak MJPM, Kompier MAJ. Getting better, but not well: a 1.5 year follow-up of cognitive performance and cortisol levels in clinical and non-Clinical burnout. Biol Psychol [Internet]. 2016 May 1;117:89–99. Available from: https://linkinghub.elsevier.com/retrieve/pii/S0301051116300461. 10.1016/j.biopsycho.2016.02.009 26930250

[77] Persson Asplund R, Dagöö J, Fjellström I, Niemi L, Hansson K, Zeraati F, et al. Internet-based stress management for distressed managers: results from a randomised controlled trial. Occup Environ Med [Internet]. 2018 Feb;75(2):105–13. Available from: https://oem.bmj.com/lookup/doi/10.1136/oemed-2017-104458. 10.1136/oemed-2017-104458 PMC5800342 28855344

[78] Salmela-aro K, Näätänen P, Nurmi J erik. The role of work-related personal projects during two burnout interventions: a longitudinal study. Work Stress [Internet]. 2004 Jul;18(3):208–30. Available from: http://www.tandfonline.com/doi/abs/10.1080/02678370412331317480.

[79] Salomonsson S, Santoft F, Lindsäter E, Ejeby K, Ingvar M, Ljótsson B, et al. Effects of cognitive behavioural therapy and return‐to‐work intervention for patients on sick leave due to stress‐related disorders: Results from a randomized trial. Scand J Psychol [Internet]. 2020 Apr 6;61(2):281–9. Available from: https://onlinelibrary.wiley.com/doi/10.1111/sjop.12590. 10.1111/sjop.12590 31691305

[80] Santoft F, Salomonsson S, Hesser H, Lindsäter E, Ljótsson B, Lekander M, et al. Mediators of change in cognitive behavior therapy for clinical burnout. Behav Ther [Internet]. 2019 May;50(3):475–88. Available from: https://linkinghub.elsevier.com/retrieve/pii/S0005789418301084. 10.1016/j.beth.2018.08.005 31030867

[81] Sandahl C, Lundberg U, Lindgren A, Rylander G, Herlofson J, Nygren Å, et al. Two forms of group therapy and individual treatment of work-related depression: a one-year follow-up study. Int J Group Psychother [Internet]. 2011 Oct 25;61(4):538–55. Available from: https://www.tandfonline.com/doi/full/10.1521/ijgp.2011.61.4.538. 10.1521/ijgp.2011.61.4.538 21985258

[82] Schene AH, Koeter MWJ, Kikkert MJ, Swinkels JA, McCrone P. Adjuvant occupational therapy for work-related major depression works: randomized trial including economic evaluation. Psychol Med [Internet]. 2007 Mar 20;37(03):351. Available from: http://www.journals.cambridge.org/abstract_S0033291706009366. 10.1017/S0033291706009366 17112401

[83] Stenlund T, Ahlgren C, Lindahl B, Burell G, Steinholtz K, Edlund C, et al. Cognitively oriented behavioral rehabilitation in combination with qigong for patients on long-term sick leave because of burnout: REST—a randomized clinical trial. Int J Behav Med [Internet]. 2009 Sep 16;16(3):294–303. Available from: http://link.springer.com/10.1007/s12529-008-9011-7. 10.1007/s12529-008-9011-7 19148765

[84] Stenlund T, Nordin M, Järvholm L. Effects of rehabilitation programmes for patients on long-term sick leave for burnout: a 3-year follow-up of the REST study. J Rehabil Med [Internet]. 2012;44(8):684–90. Available from: http://www.medicaljournals.se/jrm/content/?doi=10.2340/16501977-1003. 10.2340/16501977-1003 22729797

[85] Ugwoke SC, Eseadi C, Onuigbo LN, Aye EN, Akaneme IN, Oboegbulem AI, et al. A rational-emotive stress management intervention for reducing job burnout and dysfunctional distress among special education teachers. Medicine [Internet]. 2018 Apr 1;97(17):e0475. Available from: https://journals.lww.com/00005792-201804270-00036. 10.1097/MD.0000000000010475 PMC5944538 29703004

[86] van der Klink JJL. Reducing long term sickness absence by an activating intervention in adjustment disorders: a cluster randomised controlled design. Occup Environ Med [Internet]. 2003 Jun 1;60(6):429–37. Available from: https://oem.bmj.com/lookup/doi/10.1136/oem.60.6.429. 10.1136/oem.60.6.429 PMC1740545 12771395

[87] van Noppen P, van Dun K, Depestele S, Verstraelen S, Meesen R, Manto M. Transcranial direct current stimulation and attention skills in burnout patients: a randomized blinded sham-controlled pilot study. F1000Res [Internet]. 2020 Feb 14;9:116. Available from: https://f1000research.com/articles/9-116/v1. 10.12688/f1000research.21831.1 PMC7737710 33363715

[88] Verkuil B, Brosschot JF, Korrelboom K, Reul-Verlaan R, Thayer JF. Pretreatment of worry enhances the effects of stress management therapy: a randomized clinical trial. Psychother Psychosom [Internet]. 2011 Apr;80(3):189–90. Available from: https://www.karger.com/Article/FullText/320328. 10.1159/000320328 21389758

[89] Willert MV, Thulstrup AM, Bonde JP. Effects of a stress management intervention on absenteeism and return to work – results from a randomized wait-list controlled trial. Scand J Work Environ Health [Internet]. 2011 May;37(3):186–95. Available from: http://www.sjweh.fi/show_abstract.php?abstract_id=3130. 10.5271/sjweh.3130 21057736

[90] Willert MV, Thulstrup AM, Hertz J, Bonde JP. Changes in stress and coping from a randomized controlled trial of a three-month stress management intervention. Scand J Work Environ Health [Internet]. 2009 Mar;35(2):145–52. Available from: http://www.sjweh.fi/show_abstract.php?abstract_id=1313. 10.5271/sjweh.1313 19308298

[91] Zielhorst T, van den Brule D, Visch V, Melles M, van Tienhoven S, Sinkbaek H, et al. Using a digital game for training desirable behavior in cognitive–behavioral therapy of burnout syndrome: a controlled study. Cyberpsychol Behav Soc Netw [Internet]. 2015 Feb 1;18(2):101–11. Available from: http://www.liebertpub.com/doi/10.1089/cyber.2013.0690. 10.1089/cyber.2013.0690 25684611

[92] Adina M, Vesa Ștefan C, Nirestean A. Burnout syndrome: therapeutic approach with beneficial effects on personality and quality of life. Altern Ther Health Med [Internet]. 2021 Nov;27(6):8–14. Available from: http://www.ncbi.nlm.nih.gov/pubmed/33789249. 33789249

[93] Ekstedt M, Söderström M, Åkerstedt T. Sleep physiology in recovery from burnout. Biol Psychol [Internet]. 2009 Dec;82(3):267–73. Available from: https://linkinghub.elsevier.com/retrieve/pii/S030105110900163X. 10.1016/j.biopsycho.2009.08.006 19699775

[94] Ekvall Hansson E. Multidisciplinary program for stress-related disease in primary health care. J Multidiscip Healthc [Internet]. 2009 May;2:61. Available from: http://www.dovepress.com/multidisciplinary-program-for-stress-related-disease-in-primary-health-peer-reviewed-article-JMDH. 10.2147/jmdh.s5298 PMC3004555 21197348

[95] Firth-Cozens J, Hardy GE. Occupational stress, clinical treatment and changes in job perceptions. J Occup Organ Psychol [Internet]. 1992 Jun;65(2):81–8. Available from: https://onlinelibrary.wiley.com/doi/10.1111/j.2044-8325.1992.tb00486.x.

[96] Grigorescu S, Cazan AM, Rogozea L, Grigorescu DO. Original targeted therapy for the management of the burnout syndrome in nurses: an innovative approach and a new opportunity in the context of predictive, preventive and personalized medicine. EPMA Journal [Internet]. 2020 Jun 6;11(2):161–76. Available from: http://link.springer.com/10.1007/s13167-020-00201-6. 10.1007/s13167-020-00201-6 PMC7272529 32549915

[97] Kjellgren A, Buhrkall H. Preventing sick-leave for sufferers of high stress-load and burnout syndrome: a pilot study combining psychotherapy and the flotation tank Torsten Norlander Karolinska Institutet [Internet]. Article in International Journal of Psychology and Psychological Therapy. 2011.

[98] Lindström C, Åman J, Anderzén-Carlsson A, Lindahl Norberg A. Group intervention for burnout in parents of chronically ill children - a small-scale study. Scand J Caring Sci [Internet]. 2016 Dec 1;30(4):678–86. Available from: https://onlinelibrary.wiley.com/doi/10.1111/scs.12287. 10.1111/scs.12287 26395446

[99] Meesters Y, Horwitz, van Velzen. Day treatment of patients with severe work-related complaints. Psychol Res Behav Manag [Internet]. 2012 May;5:57. Available from: http://www.dovepress.com/day-treatment-of-patients-with-severe-work-related-complaints-peer-reviewed-article-PRBM. 10.2147/PRBM.S31032 PMC3378180 22715319

[100] Millet P. Integrating horticulture into the vocational rehabilitation process of individuals with exhaustion syndrome (burnout): a pilot study. Intern J Dis Manag [Internet]. 2008 Sep 1;3(2):39–53. Available from: https://www.cambridge.org/core/product/identifier/S1833855000000256/type/journal_article.

[101] Mommersteeg PMC, Keijsers GPJ, Heijnen CJ, Verbraak MJPM, van Doornen LJP. Cortisol deviations in people with burnout before and after psychotherapy: a pilot study. Health Psychology [Internet]. 2006 Mar;25(2):243–8. Available from: http://doi.apa.org/getdoi.cfm?doi=10.1037/0278-6133.25.2.243. 10.1037/0278-6133.25.2.243 16569117

[102] Mommersteeg PMC, Heijnen CJ, Verbraak MJPM, van Doornen LJP. A longitudinal study on cortisol and complaint reduction in burnout. Psychoneuroendocrinology [Internet]. 2006 Aug;31(7):793–804. Available from: https://linkinghub.elsevier.com/retrieve/pii/S0306453006000527. 10.1016/j.psyneuen.2006.03.003 16698185

[103] Orosz A, Federspiel A, Eckert A, Seeher C, Dierks T, Tschitsaz A, et al. Exploring the effectiveness of a specialized therapy programme for burnout using subjective report and biomarkers of stress. Clin Psychol Psychother [Internet]. 2021 Jul 15;28(4):852–61. Available from: https://onlinelibrary.wiley.com/doi/10.1002/cpp.2539. 10.1002/cpp.2539 33283948

[104] Pálsdóttir AM, Grahn P, Persson D. Changes in experienced value of everyday occupations after nature-based vocational rehabilitation. Scand J Occup Ther [Internet]. 2013 Sep 17;21(1):1–11. Available from: http://www.tandfonline.com/doi/full/10.3109/11038128.2013.832794. 10.3109/11038128.2013.832794 24041155

[105] Sahlin E, Ahlborg G, Matuszczyk J, Grahn P. Nature-based stress management course for individuals at risk of adverse health effects from work-related stress—effects on stress related symptoms, workability and sick leave. Int J Environ Res Public Health [Internet]. 2014 Jun 23;11(6):6586–611. Available from: http://www.mdpi.com/1660-4601/11/6/6586. 10.3390/ijerph110606586 PMC4078597 25003175

[106] Sahlin E, Ahlborg G, Tenenbaum A, Grahn P. Using nature-based rehabilitation to restart a stalled process of rehabilitation in individuals with stress-related mental illness. Int J Environ Res Public Health [Internet]. 2015 Feb 9;12(2):1928–51. Available from: http://www.mdpi.com/1660-4601/12/2/1928. 10.3390/ijerph120201928 PMC4344702 25671775

[107] Steensma H, Heijer M den, Stallen V. Research note: effects of resilience training on the reduction of stress and depression among Dutch workers. Int Q Community Health Educ [Internet]. 2007 Jul 25;27(2):145–59. Available from: http://journals.sagepub.com/doi/10.2190/IQ.27.2.e. 10.2190/IQ.27.2.e 18364303

[108] Thomas TE, Eyal R, Menchavez F, Mocci T, Goldblatt G, Lanoff J, et al. Reducing workplace absenteeism caused by work stress in a health maintenance organization department of psychiatry. Perm J [Internet]. 2020 Mar;24(1). Available from: http://www.thepermanentejournal.org/doi/10.7812/TPP/19.027. 10.7812/TPP/19.027 PMC6907906 31852041

[109] van de Leur JC, Buhrman M, Åhs F, Rozental A, Jansen GB. Standardized multimodal intervention for stress-induced exhaustion disorder: an open trial in a clinical setting. BMC Psychiatry [Internet]. 2020 Dec 5;20(1):526. Available from: https://bmcpsychiatry.biomedcentral.com/articles/10.1186/s12888-020-02907-3. 10.1186/s12888-020-02907-3 PMC7643309 33153461

[110] Hätinen M, Kinnunen U, Mäkikangas A, Kalimo R, Tolvanen A, Pekkonen M. Burnout during a long-term rehabilitation: comparing low burnout, high burnout – benefited, and high burnout – not benefited trajectories. Anxiety Stress Coping [Internet]. 2009 May;22(3):341–60. Available from: http://www.tandfonline.com/doi/abs/10.1080/10615800802567023. 10.1080/10615800802567023 19283645

[111] Kinnunen SM, Puolakanaho A, Tolvanen A, Mäkikangas A, Lappalainen R. Does mindfulness-, acceptance-, and value-based intervention alleviate burnout?—A person-centered approach. Int J Stress Manag [Internet]. 2019 Feb 1;26(1):89–101. Available from: http://doi.apa.org/getdoi.cfm?doi=10.1037/str0000095.

[112] van der Meulen RT, Valentin S, Bögels SM, de Bruin EI. Mindfulness and self-compassion as mediators of the Mindful2Work Training on perceived stress and chronic fatigue. Mindfulness (N Y) [Internet]. 2021 Apr 20;12(4):936–46. Available from: http://link.springer.com/10.1007/s12671-020-01557-6.

[113] Engebretsen KM. Suffering without a medical diagnosis. A critical view on the biomedical attitudes towards persons suffering from burnout and the implications for medical care. J Eval Clin Pract. 2018 Oct 1;24(5):1150–7. 10.1111/jep.12986 30003618

[114] Varga S. Defining mental disorder. Exploring the “natural function” approach. Philosophy, Ethics, and Humanities in Medicine [Internet]. 2011;6(1):1. Available from: 10.1186/1747-5341-6-1. 10.1186/1747-5341-6-1 PMC3031189 21255405

[115] Pilgrim D (2007). The survival of psychiatric diagnosis. Soc Sci Med.

[116] Maj M. Why the clinical utility of diagnostic categories in psychiatry is intrinsically limited and how we can use new approaches to complement them. Vol. 17, World Psychiatry. Blackwell Publishing Ltd; 2018. p. 121–2. 10.1002/wps.20512 PMC5980436 29856539

[117] Kupfer DJ, First M, Regier DA. A Research Agenda for DSM-V. In 2002.

[118] Kendler KS. Reviews and overviews toward a philosophical structure for psychiatry [Internet]. Vol. 162, Am J Psychiatry. 2005. Available from: http://ajp.psychiatryonline.org. 10.1176/appi.ajp.162.3.433 15741457

[119] Peters A, McEwen BS, Friston K. Uncertainty and stress: why it causes diseases and how it is mastered by the brain. Prog Neurobiol [Internet]. 2017;156:164–88. Available from: 10.1016/j.pneurobio.2017.05.004. 10.1016/j.pneurobio.2017.05.004 28576664

[120] Hofmann SG, Curtiss J. A complex network approach to clinical science. Eur J Clin Invest [Internet]. 2018 Aug;48(8):e12986. Available from: https://onlinelibrary.wiley.com/doi/10.1111/eci.12986. 10.1111/eci.12986 29931701

[121] Borsboom D. A network theory of mental disorders. World Psychiatry [Internet]. 2017 Feb;16(1):5–13. Available from: https://onlinelibrary.wiley.com/doi/10.1002/wps.20375. 10.1002/wps.20375 PMC5269502 28127906

[122] Hayes SC, Hofmann SG, Ciarrochi J. A process-based approach to psychological diagnosis and treatment: the conceptual and treatment utility of an extended evolutionary meta model. Clin Psychol Rev [Internet]. 2020 Dec;82:101908. Available from: https://linkinghub.elsevier.com/retrieve/pii/S0272735820300969. 10.1016/j.cpr.2020.101908 PMC7680437 32932093

[123] Hayes SC, Ciarrochi J, Hofmann SG, Chin F, Sahdra B. Evolving an idionomic approach to processes of change: towards a unified personalized science of human improvement. Behav Res Ther [Internet]. 2022 Sep;156:104155. Available from: https://linkinghub.elsevier.com/retrieve/pii/S0005796722001267. 10.1016/j.brat.2022.104155 35863243

[124] Lundgren T, Parling T (2016). Swedish acceptance and action questionnaire (SAAQ): a psychometric evaluation. Cogn Behav Ther.

