# Supplementary material for: Psychological Treatment of Exhaustion Due to Persistent Non-Traumatic Stress: A Scoping Review
Source: Int J Behav Med. 2023 Jun 12;31(2):175–91. doi: 10.1007/s12529-023-10185-y (PMC11001660; doi:10.1007/s12529-023-10185-y)
Supplement: Supplementary file 1 — Supplementary file1 (PDF 824 KB) [file 12529_2023_10185_MOESM1_ESM.pdf]

SUPPLEMENTARY MATERIAL

Psychological treatment of Exhaustion due to Persistent Non-traumatic Stress: A Scoping Review

Preferred Reporting Items for Systematic reviews and Meta-Analyses extension for Scoping Reviews (PRISMA-ScR) Checklist

SECTION	ITEM	PRISMA-ScR CHECKLIST ITEM	REPORTED ON PAGE #
**TITLE**			
Title	1	Identify the report as a scoping review.	
**ABSTRACT**			
Structured summary	2	Provide a structured summary that includes (as applicable): background, objectives, eligibility criteria, sources of evidence, charting methods, results, and conclusions that relate to the review questions and objectives.	
**INTRODUCTION**			
Rationale	3	Describe the rationale for the review in the context of what is already known. Explain why the review questions/objectives lend themselves to a scoping review approach.	
Objectives	4	Provide an explicit statement of the questions and objectives being addressed with reference to their key elements (e.g., population or participants, concepts, and context) or other relevant key elements used to conceptualize the review questions and/or objectives.	
**METHODS**			
Protocol and registration	5	Indicate whether a review protocol exists; state if and where it can be accessed (e.g., a Web address); and if available, provide registration information, including the registration number.	
Eligibility criteria	6	Specify characteristics of the sources of evidence used as eligibility criteria (e.g., years considered, language, and publication status), and provide a rationale.	
Information sources*	7	Describe all information sources in the search (e.g., databases with dates of coverage and contact with authors to identify additional sources), as well as the date the most recent search was executed.	
Search	8	Present the full electronic search strategy for at least 1 database, including any limits used, such that it could be repeated.	
Selection of sources of evidence	9	State the process for selecting sources of evidence (i.e., screening and eligibility) included in the scoping review.	
Data charting process	10	Describe the methods of charting data from the included sources of evidence (e.g., calibrated forms or forms that have been tested by the team before their use, and whether data charting was done independently or in duplicate) and any processes for obtaining and confirming data from investigators.	
Data items	11	List and define all variables for which data were sought and any assumptions and simplifications made.	
Critical appraisal of individual sources of evidence§	12	If done, provide a rationale for conducting a critical appraisal of included sources of evidence; describe the methods used and how this information was used in any data synthesis (if appropriate).	
Synthesis of results	13	Describe the methods of handling and summarizing the data that were charted.	

SECTION	ITEM	PRISMA-ScR CHECKLIST ITEM	REPORTED ON PAGE #
**RESULTS**			
Selection of sources of evidence	14	Give numbers of sources of evidence screened, assessed for eligibility, and included in the review, with reasons for exclusions at each stage, ideally using a flow diagram.	
Characteristics of sources of evidence	15	For each source of evidence, present characteristics for which data were charted and provide the citations.	
Critical appraisal within sources of evidence	16	If done, present data on critical appraisal of included sources of evidence (see item 12).	
Results of individual sources of evidence	17	For each included source of evidence, present the relevant data that were charted that relate to the review questions and objectives.	
Synthesis of results	18	Summarize and/or present the charting results as they relate to the review questions and objectives.	
**DISCUSSION**			
Summary of evidence	19	Summarize the main results (including an overview of concepts, themes, and types of evidence available), link to the review questions and objectives, and consider the relevance to key groups.	
Limitations	20	Discuss the limitations of the scoping review process.	
Conclusions	21	Provide a general interpretation of the results with respect to the review questions and objectives, as well as potential implications and/or next steps.	
**FUNDING**			
Funding	22	Describe sources of funding for the included sources of evidence, as well as sources of funding for the scoping review. Describe the role of the funders of the scoping review.	

JBI = Joanna Briggs Institute; PRISMA-ScR = Preferred Reporting Items for Systematic reviews and Meta-Analyses extension for Scoping Reviews.
* Where *sources of evidence* (see second footnote) are compiled from, such as bibliographic databases, social media platforms, and Web sites.
PRHLFOLHKHWHRJHHRWHPHGWRDFFR[illegible] nt for the different types of evidence or data sources (e.g., quantitative and/or qualitative research, expert opinion, and policy documents) that may be eligible in a scoping review as opposed to only studies. This is not to be confused with *information sources* (see first footnote).
7KHIDPHZREHDG20DOOHDG/HDFDGFROOHDJHDGWKH-[illegible]LGDFHHIHWRWKH[illegible] process of data extraction in a scoping review as data charting.
§ The process of systematically examining research evidence to assess its validity, results, and relevance before using it to inform a decision. This term is used for items 12 and 19 instead of "risk of bias" (which is more applicable to systematic reviews of interventions) to include and acknowledge the various sources of evidence that may be used in a scoping review (e.g., quantitative and/or qualitative research, expert opinion, and policy document).

From: Tricco AC, Lillie E, Zarin W, O'Brien KK, Colquhoun H, Levac D, et al. PRISMA Extension for Scoping Reviews (PRISMAScR): Checklist and Explanation. Ann Intern Med. 2018;169:467[illegible]473. doi: 10.7326/M18-0850.

Final search terms

Search terms in the title/abstract are identical across all three databases. The MeSH differs somewhat between the databases due to differences in cataloging.

PUB MED

(((burnout, professional[MeSH Terms]) OR (adjustment disorders[MeSH Terms]) OR (neurasthenia[MeSH Terms]) OR (non-traumatic stress[Title/Abstract]) OR (non traumatic stress[Title/Abstract]) OR (persistent stress[Title/Abstract]) OR (long term stress[Title/Abstract]) OR (neurasthenia[Title/Abstract]) OR (work stress[Title/Abstract]) OR (work related stress[Title/Abstract]) OR (occupational stress [Title/Abstract]) OR (burnout[Title/Abstract]) OR (exhaustion disorder[Title/Abstract]) OR (exhaustion depression[Title/Abstract]) OR (exhaustion syndrome[Title/Abstract])) AND ((psychotherapy[MeSH Terms]) OR (cognitive therapy[MeSH Terms]) OR (behavior therapy[MeSH Terms]) OR (Mind-Body Therapies[MeSH Terms]) OR (Biofeedback, Psychology[MeSH Terms]) OR (behavior therapy[Title/Abstract]) OR (behavior therapies[Title/Abstract]) OR (behavioral therapy[Title/Abstract]) OR (behavioral therapies[Title/Abstract]) OR (behavioural therapy[Title/Abstract]) OR (behavioural therapies[Title/Abstract])OR (cognitive therapy[Title/Abstract]) OR (cognitive therapies[Title/Abstract]) OR (compassion focused[Title/Abstract]) OR (acceptance and commitment [Title/Abstract]) OR (meditat*[Title/Abstract]) OR (mindful*[Title/Abstract]) OR (psychotherap*[Title/Abstract]) OR (psychological treatment[Title/Abstract]) OR (psychological therapy[Title/Abstract]) OR (psychological therapies[Title/Abstract]) OR (psychological intervention[Title/Abstract]) OR (rehabilitation[Title/Abstract]) OR (group therapy[Title/Abstract]) OR (group therapies[Title/Abstract]) OR (self-regulation training[Title/Abstract]) OR (self regulation training[Title/Abstract]) OR (psycho-education*[Title/Abstract]) OR (cognitive training[Title/Abstract]) OR (internet-based[Title/Abstract]) OR (internet intervention*[Title/Abstract]) OR (skills training[Title/Abstract]) OR (resilience training[Title/Abstract]) OR (coping skills[Title/Abstract]) OR (stress management[Title/Abstract])))

PSYCH INFO

(MM (burnout, professional OR adjustment disorders OR neurasthenia) OR AB ("non-traumatic stress" OR "non traumatic stress" OR "persistent stress" OR "long term stress" OR neurasthenia OR "work stress" OR "work related stress" OR "occupational stress" OR burnout OR "exhaustion disorder" OR "exhaustion depression" OR "exhaustion syndrome")) AND (MM (psychotherapy OR cognitive therapy OR behavior therapy OR Mind-Body Therapies OR Biofeedback, Psychology OR AB ("behavior therapy" OR "behavior therapies" OR "behavioral therapy" OR "behavioral therapies" OR "behavioural therapy" OR "behavioural therapies" OR "cognitive therapy" OR "cognitive therapies" OR "compassion focused" OR "acceptance and commitment" OR meditat* OR mindful* OR psychotherap* OR "psychological treatment" OR "psychological therapy" OR "psychological therapies" OR "psychological intervention" OR rehabilitation OR "group therapy" OR "group therapies" OR "self-regulation training" OR "self regulation training" OR "psycho-education*" OR "cognitive training" OR internet-based OR "internet intervention*" OR "skills training" OR "resilience training" OR "coping skills" OR "stress management"))

CINAHL

(MM (“burnout, professional” OR “stress, occupational” OR "adjustment disorders" OR "compassion fatigue") OR AB ("non-traumatic stress" OR "non traumatic stress" OR "persistent stress" OR "long term stress" OR neurasthenia OR "work stress" OR "work related stress" OR "occupational stress" OR burnout OR "exhaustion disorder" OR "exhaustion depression" OR "exhaustion syndrome")) AND (MM (“psychotherapy” OR “cognitive therapy” OR “cognitive therapy” OR “mindfulness” OR “meditation” OR “stress management”) OR AB ("behavior therapy" OR "behavior therapies" OR "behavioral therapy" OR "behavioral therapies" OR "behavioural therapy" OR "behavioural therapies" OR "cognitive therapy" OR "cognitive therapies" OR "compassion focused" OR "acceptance and commitment" OR meditat* OR mindful* OR psychotherap* OR "psychological treatment" OR "psychological therapy" OR "psychological therapies" OR "psychological intervention" OR rehabilitation OR "group therapy" OR "group therapies" OR "self-regulation training" OR "self regulation training" OR "psycho-education*" OR "cognitive training" OR internet-based OR "internet intervention*" OR "skills training" OR "resilience training" OR "coping skills" OR "stress management"))

Supplementary table. Complete data extraction of all the included studies (N = 60).

Authors, Year	Study location	Design, Participants (dropouts), Control Condition (n)	Age (SD), Female %	Term used for ENTS	Diagnostic label(s)	Treatment, format, treatment length, number of sessions (session length)	Performing profession	Follow-up	Outcomes	Main outcomes	Effect sizes	Summary of Secondary results and analysis
**Case studies**												
Anclair & Hiltunen, 2014 [1]	Sweden	Single case studies, 2 (0), No	46 (1), 100 %	Stress related problems	No	CBT/ACT, Individual, Case 1: 18 weeks, 18 (1h) Case 2: 17 weeks, 17 (1h)	NS	No	Primary not defined. SMBQ, behavioral measures, MADRS-S (Case 2)	Pre-post: Case 1: SMBQ: 4.86/2.80 Case 2: MADRS-S: 24/7 SMBQ: 4.27/2.63	NS	NS
Barkham & Shapiro, 1990 [2]	England	Case study, 12 (0), non-randomized active control (6)	NS	Job-related distress	Mild depression (according to BDI)	Prescriptive therapy vs Exploratory therapy, Individual, two one-hour sessions one week apart followed by a third session three months later (NS)	Clinical psychologists	6 months	Primary not defined. BDI, SLC-90R	No p-values. Describes 55 – 73 % showed within reliable and clinical improvement at 6 months depending on outcome instrument (only described in tables)	NS	NS
Gyllensten & Palmer, 2005 [3]	England	Case study, 1(0), No	28, woman	Work-place stress	No	CBT, Individual, NS, 10 (NS)	Trainee counselling therapist (first authors)	1 month	Primary not defined. DASS	Pre-1MFU: DASS scale stress = 24/0 anxiety = 10/0 depression = 24/1	NS	NS
**Between studies**												
Beck et al., 2015 [4]	Denmark	RCT, 20 (1), WLC (7)	44.8 (8.8), 80 %	Work-related stress	No	Guided imagery and music (GIM), individual, 9 weeks, 6 (NS)	"Therapist", not furtherly specified	6 months	Primary not defined. PSS, POMS-37, KSQ, GAD-7, MDI, WHO-5 Well-Being Index (even more measures), testosterone, salivary cortisol, melatonin, Sick leave data.	Pre-post: WHO-5 ($p < .05$) POMS-37 ($p < .01$) GAD-7 ($p < .01$)	Pre-post: WHO-5 d = 1.37 POMS-37 d = 0.90 GAD-7 d = 0.75	Between pre-post salivary cortisol ($p < .05$) with a medium effect size (d = 0.43).
Bertoch et al., 1989 [5]	USA	RCT, 30 (0), No treatment (15)	38.1 (8.3), 60 %	Occupational stress	No	Holistic treatment, Group, NS, 12 (2 h)	Clinical psychologists	No	Primary not defined. SCSI, DSP, OSI, TSM	Post: SCSI ($p < .01$) DSP ($p < .05$) OSI ($p < .01$) TSM: ($p < .001$)	Post (ES not defined): SCSI: ES = 1.22 DSP: ES = 1.00 OSI: ES = 0.75 TSM: ES = 1.10	NS

Beutel et al., 2006 [6]	Germany	Quasi random ABAB design, 279 (13 % in the intervention group), TAU (135)	39 (NS), 60 %	Occupational stress	No	Psychodynamic, Group, 4 weeks, 8 (90 min).	Psychotherapist	No	Primary not defined. Vocational attitudes using non standardized scale, Treatment satisfaction,	Post: Treatment satisfaction ($p < .05$)	NS	NS
Buxton et al., 2020 [7]	Germany	RCT (pilot), 82 (5), psycho-pharmacological consultation waitlist condition (PCC) (34)	45.5 (8.5), 45 %	Work-related stress and resulting depression	MDD (DSM-IV)	MDT-DH (+ CBT elements), Group, 8 weeks, NS (150 h of treatment in total).	Two trained clinicians (clinical psychologist and psychiatrist) with extensive mindfulness experience (e.g., daily mindfulness practice, participation in retreats), and two certified MBSR/MBCT - teachers.	8 months	**Primary:** BDI-II **Secondary:** KIMS-D, WAI	Pre-post: Depression ($p < .001$) KIMS-D ($p < .001$) WAI ($p < .001$)	Pre-post: Depression: $g_{Hedges} = 1.80$ KIMS-D: $g_{Hedges} = 1.62$ WAI: $g_{Hedges} = 1.40$	NS
Dalgaard et al., 2017 [8]	Denmark	Prospective RCT, 163 (NS), Treatment Three arms: Treatment (58)/ Assessment (56)/No offers (49)	45.0 (NS), 74 %	Work related stress	Work related ICD-condition F43,2–F43,9 (but not PTSD) or mild depression (F32.0)	Work focused CBT, Individual, 16 weeks, 6 (1h)	Psychologist	10 months after baseline	**Primary:** PSS-10, GHQ-30 **Secondary:** BNSQ, CFQ	No significant differences between groups.	NS	NS
de Vente et al., 2008 [9]	The Netherlands	RCT, 82 (14), Three arms: individual SMT (28), Group SMT (28) and CAU (26)	41.4 (9.7), 64 %	Work-related stress	No. The presence of symptoms of neurasthenia, work-related etiology	SMT, Group or Individual, 16 weeks, 12 (NS)	Twelve experienced therapists with a master's degree in clinical psychology.	4, 7 and 10 months	Primary not defined. MBI-GS, CIS (General fatigue), DASS, Absenteeism (self-report)	No significant differences between groups.	NS	NS
Eklund & Erlandsson, 2011 [10–14]	Sweden	Quasi-experimental clinical trial, 84 (6), CAU (42)	45.5 (14.8), 100 %	Work-related stress	Depression, ED (F43.8)	Occupational therapy (analysis and behavior change of everyday behaviors), Group, 16 weeks, 23 (2,5 h)	Licensed occupational therapist	12 month and 3-4 years	Primary not defined. PSS, HADS, MANSA, PM scale, RTW	No significant differences between groups. No between difference in sick leave at 3-4 YFU (Eklund, 2017).	NS	No relationship between changes in perceptions of the work environment and outcomes post (Wästberg et al., 2016)
Ezenwaji et al., 2019 [15]	Nigeria	RCT, 52 (0), no-intervention control group (26)	20.0 (1.8), 55 %	Burnout	No	Rational-emotive behavior therapy (REBT), Group, 12 weeks, 24 (3 h)	Three of the authors with expertise in psychological coaching and REBT theory. Clinical education level not described.	3 months	Primary not defined. PSS, OLBI-S	Post and 3MFU: OLBI-S exhaustion and disengagement ($p < .001$)	Post: Exhaustion: $\eta^2p = .49$ Disengagement: $\eta^2p = .82$ 3MFU: Exhaustion: $\eta^2p = .44$ Disengagement: $\eta^2p = .72$	NS

Ezeudu et al., 2020 [16]	Nigeria	RCT, 30 (0), TAU (15)	21.4 (4.7), 63 %	Burnout	No	REBT, NS, 10 weeks, NS	NS	No	Primary not defined. OLBI-S	Pre-post: OLBI-S (p < .001)	Pre-post: OLBI-S: η^2p = .597	NS
Fang et al., 2021 [17]	USA	RCT, 66 (6), Three arms: Reward (17) / Approach (30) / Control (19)	27.1 (3.0), 76.6 %	Burnout	No	Behavioral activation (BA), Individual (via phone), 1 week, 1 (NS)	One therapist (advanced doctoral student with extensive training in CBTs and specialized training in BA).	1 week	**Primary:** SBI<br><br>**Secondary:** IDAS, BADS, EROS, PM scale, PANAS	Interaction between group and time SBI (p < .05)	At follow-up: Approach vs control d = 1.11	Doctoral students in both intervention conditions reported more behavioral activation (BADS) over time, while the students in the control condition reported decreases in positive affect (PANAS) over time.
Finnes et al., 2017 [18,19]	Sweden	RCT, 352 (23), Four arms: ACT (89). ACT+WDI (88), WDI (87), TAU (88)	46.3 (8.9), 79 %	Stress-related ill-health	ED (Swedish ICD-10: F 43.8)	ACT, WDI, ACT+WDI combination, Individual, 3 months, 6 (NS)	Clinical licensed psychologists delivered ACT. The therapists delivering WI were licensed clinical psychologists, a behavioral therapist, and a nurse specialized in psychiatric care	9 months	**Primary:** Sick-leave (net SA-days), WAI<br>**Secondary:** WSAS, SWLS, KEDS, HADS	No significant difference between the groups from pre-post in SA-days or WI.<br>Pre-post: KEDS and HADSdep (.01 < p < .05) in ACT and ACT+WDI	Pre-post: KEDS and HADSdep d = 0.26-0.46 Differences did not remain at follow-up	For ED adding WDI to ACT seems to reduce healthcare costs, while WDI as a stand-alone intervention seems to reduce welfare costs.
Firth & Shapiro, 1986 [20]	England	Crossover design, 46 (6), No	40.4 (NS), 43 %	Job-related distress	No	Prescriptive therapy (CBT) + Exploratory therapy (psychodynamic), Individual, 21 weeks, 16 (NS)	Clinical psychologists	3 months	Primary not defined.<br><br>PSE, WSAS	Prescriptive more effective than Exploratory (p < .001) in reducing symptoms at mid-treatment (i.e., at the end of the first of two received treatments)	NS	NS
Glasscock et al., 2018 [21]	Denmark	Prospective RCT, 137 (25), No intervention (80)	45.0 (NS), 84 %	Work related stress.	ICD-10 code F43.2–F43.9, but not PTSD) or mild depression (F32.0)	CBT, Individual, 4 months maximum, 6 (60 min)	Psychologist	4 and 10 months	Primary not defined. PSS, GHQ, RTW.	4MFU:<br>PSS (p < .011)<br>GHQ (p < .018)<br>No differences in RTW	4MFU:<br>PSS d = -0.51<br>GHQ d = -0.54	Symptom differences did not remain at 10MFU.

Grahn et al., 2017 [22]	Sweden	Prospective quasi-experimental study, comparing lengths of intervention, 106 (0), Three arms: 8 weeks/12 weeks/24 weeks	45.4 (NS), 83 %	Severe stress and/or depression	Reaction to severe stress (ICD-10 F43.8/F43.9) or MDD (F32.0/F32.1)	Nature based rehabilitation + meetings with a psychotherapist, Group, 8/12/24, 32/48/96 (3,5 h).	Multimodal team of: physiotherapist, psychotherapist, psychiatrist, landscape architect etc.	12 months after baseline	**Primary**: RTW<br>**Secondary**: OSA, Mastery scale, SoC-29	12MFU: RTW higher for 24-week program compared to 12 and 8-week ($p < .05$). RTW higher for 12-week program compared to 8-week ($p < .05$).	12MFU: RTW<br>8 weeks: g_{Hedges} = 0.85<br>12 weeks: g_{Hedges} = 1.22<br>24 weeks: g_{Hedges} = 1.49	No significant differences between interventions regarding symptoms.
Grensman et al., 2018 [23]	Sweden	RCT, 94 (14), Three arms: Traditional yoga (32)/MBCT (31)/ CBT (31)	44.0 (3.2), 88 %	Burnout	ED	Traditional Yoga/MBCT/CBT, Group, 20 weeks, NS, (1-1,5 h)	Physician (Yoga, MBSR) psychotherapist (CBT).	No	Primary not defined. SWED-QUAL	No significant differences between groups.	Pre-post, within: d = 0.5-1.12 on various subscales of SWED-QUAL for all treatments	NS
Hätinen et al., 2007 [24]	Finland	Quasi-experimental clinical trial, 64 (9), Three arms: Traditional (32)/Participatory (20)/WLC (12)	49.3 (6.5), 100 %	Burnout	No	Traditional vs participatory intervention (generic coping), Individual and group, 12+5 days, NS	MDs, licensed psychologist and licensed physiotherapist	No	Primary not defined. MBI-GS (exhaustion, cynicism, reduced professional efficacy), *Time pressure*, *Job control*, *Workplace-climate*	Group x Time interaction MBI-GS ($p < .05$) in favor of participatory intervention pre-post<br>Within pre-post: Exhaustion ($p < .001$) and cynicism ($p < .05$) for participatory intervention.<br>No effects on MBI-GS for traditional intervention.<br>No significant effects at follow-up.	Post: MBI-GS $\eta2$ = .13<br>Pre-post: Within participatory exhaustion $\eta2$ = .60<br>Within participatory cynicism $\eta2$ = .27	Effects of exhaustion mediated by workplace climate and job control in participatory group.
Igbokwe et al., 2019 [25]	Nigeria	RCT, 96 (0) No intervention (48)	21.2 (1.6), 79 %	Burnout	No	REBT, NS, 10 weeks, 20 (80 min)	The authors, not furtherly specified	3 months	**Primary:** OLBI-S (Exhaustion, Disengagement)	Pre-3MFU:<br>Exhaustion ($p < .001$)<br>Disengagement ($p < .001$)	Pre-3MFU:<br>Exhaustion: η^2p = .601<br>Disengagement: η^2p = .837	NS
Lappalainen et al, 2013 [26]	Finland	RCT (pilot), 24 (1), No intervention (12)	43.1 (7.6), 0 %	Stress-related psychological problems.	No	CBT/ACT, Group/internet/app, 12 weeks, NS	Psychologist	6 months	**Primary:** BDI, SCL-90 (GSI), BBI-15<br>**Secondary:** QoL (VAS), Working ability (VAS) AAQ-2, ERI	No significant between differences BDI and SCL-90<br>6MFU:<br>Working ability ($p = .016$)	6MFU:<br>Working ability d = 0.21	Intervention within:<br>BDI ($p = .001$)<br>GSI ($p = .001$)<br>BBI-15 ($p = .006$)<br>Working ability ($p = .01$)
Lindsäter et al., 2018 [27,28]	Sweden	RCT, 100 (9), WLC (50)	46.2 (8.8), 85 %	Chronic Stress	Adjustment disorder (DSM-IV) and (Swedish ICD-10: F 43.8)	CBT, Internet-based, 12 weeks, weekly online support	Psychologist	6 months	**Primary:** PSS<br>**Secondary:** SMBQ, ISI	Pre-post:<br>PSS ($p < .001$)<br>SMBQ ($p < .001$)<br>ISI p.001<br>GAD-7 ($p < .001$)<br>MADRS-S ($p < .001$)	Pre-post:<br>PSS: d= 1.09<br>SMBQ: d= 1.09<br>ISI: d= 1.05<br>GAD-7: d= 0.57<br>MADRS-S: d= 0.67	Reduction in insomnia symptoms mediated the effect of iCBT on symptoms of stress and exhaustion ($p < .001$)

Malmberg Gavelin et al., 2015 [29–32]	Sweden	RCT, 132 (31), Three arms: MMR+Cognitive training (44)/MMR+Aerobic training (47)/ MMR (41)	43.4 (8.5), 84 %	Exhaustion disorder	ED (Swedish ICD-10: F 43.8)	Group CBT, rehabilitation meetings, spinning and cognitive training, 24, CBT Group = 22 sessions (3 h each) Spinning = 40 min session, three times a week for 12 weeks Cognitive training (from home) = 15-20 min session, three times a week for 12 weeks	Licensed psychologists and physiotherapists	12 months	**Primary:** Cognitive performance (Cognitive tests). **Secondary:** Burnout, depression, anxiety and fatigue; work ability and aerobic capacity	Pre-post: VO2max and episodic memory performance for MMR+Aerobic (Eskilsson et al., 2017). Pre-12MFU cognitive performance ($p < .02$) for cognitive training (Gavelin Malmberg et al., 2018)	Pre-12MFU: cognitive performance d = 0.35	For psychological health and work ability, no additional effects of cognitive or aerobic training were found relative to multi-modal stress rehabilitation alone.
Netterstrøm & Bech, 2010 [33]	Denmark	Controlled trial, 107 (10), Two sessions with a specialist in occupational medicine (34)	44.8 (NS), 60 %	Work-related stress induced adjustment disorder	Adjustment disorder	Generic stress treatment, Individual, 4 months, on average 6 (1-2 h)	Authors (GPs)	12 and 24 months	Primary not defined. MDI, overall functioning Stress Clinic General-wellbeing questionnaire, RTW	No significant differences on follow-up.	NS	NS
Oloidi et al., 2022 [34]	Nigeria	RCT, 35 (0), WLC (17)	23.0 (2.5), 54 %	Burnout	No	Online REBT, Individual online sessions, 6 weeks, 14 session (60 min)	NS	6 weeks	Primary not defined. OLBI-S	Interaction group x time pre vs follow-up OLBI-S ($p < .001$)	Pre-follow-up OLBI-S $\eta^2 p = .63$	NS
Oosterholt et al., 2012 [35]	The Netherlands	Matched controlled trial, 32 (0), Healthy control (16)	40.7 (10.5), 50 %	Burnout	Undifferentiated somatoform disorder with the addition of work-related causes (DSM-IV-TR)	CBT, Individual, average 10 weeks, average 11 sessions (45 min)	Professional clinical psychologists	No	Primary not defined. UBOS, SCL-90-R, CFQ, Cognitive tests (2-back-task, Sustained-attention-to-response test, Matching task)	Pre-post: UBOS emotional exhaustion ($p < .05$) SCL-90-R ($p < .05$)	Pre-post: UBOS emotional exhaustion $\eta^2 = .15$ SCL-90-R $\eta^2 = .19$	Between pre-post Matching task ($p < .001$, $\eta^2 = .68$)
Oosterholt et al., 2016[36]	The Netherlands	Matched controlled trial, 93 (35), Three arms: clinical burnout (33)/non-clinical burnout (30)/ healthy control (30)	39.7 (11.5), 46 %	Clinical Burnout	Undifferentiated somatoform disorder with the addition of work-related causes (DSM-IV-TR)	CBT, Individual, average 14 sessions (NS)	Professional clinical psychologists	18 months	Primary not defined. UBOS, SCL-90-R, CFQ, Cognitive tests (STOP-IT, Flanker Task, Matching Task), cortisol levels in saliva	Pre-18MFU: UBOS emotional exhaustion ($p < .001$) UBOS cynicism ($p < .01$) UBOS personal efficacy ($p < .001$) SCL-90-R ($p < .001$) CFQ ($p < .001$)	Pre-18MFU: UBOS emotional exhaustion $\eta^2 = .51$ UBOS cynicism $\eta^2 = .17$ UBOS personal efficacy $\eta^2 = .43$ SCL-90-R $\eta^2 = .31$ CFQ $\eta^2 = .37$	No significant findings on cortisol levels between groups.

Persson Asplund et al., 2018 [37]	Sweden	RCT, 117 (15), Attention control group (58)	46.9 (8.3), 67 %	Work-related stress, adjustment disorder	Adjustment disorder (ICD-10)	CBT (incl. 3rd wave), internet-based, 8 weeks, 8 modules	Psychologists and supervised master-level psychology students who were trained to perform feedback according to a standardized manual.	6 months	**Primary:** PSS-14 **Secondary:** SMBQ, MADRS-S, ISI, AUDIT, WEMS, TiC-P	Pre-post: PSS-14 ($p < .001$) Pre-6MFU: PSS-14 ($p < .01$)	Pre-post: PSS-14 d = 0.74 Pre-6MFU: PSS-14 d = 0.74	Significant between pre-post: SMBQ (d = 0.95), ISI (d = 0.34), maintained at 6MFU.<br><br>Significant between pre-post: MADRS-S (d = 0.86) and WEMS (d = 0.59), not maintained at 6MFU
Salmela-Aro et al., 2004 [38]	Finland	RCT, 98 (6), Three arms: Psychoanalytic (32)/ Experiential (32)/Control (32)	47.8 (6.8), 73 %	Burnout	No	Psychoanalytic vs Experiential, Group, 10 months, 16 (one day)	Therapists with at least 10 years of experience	No	Primary not defined. Personal project analysis inventory (PPA) , Bergen Burnout Indicator (BBI)	Pre-post: BBI ($p < .01$)<br><br>Pre-post: decrease in work related projects, negative, action-avoiding tendencies and project-related negative emotion (PPA $p < .001$)	NS	Between pre-post increase in project-related progress, social support and emotions (PPA $p < .001$)
Salomonsson et al., 2020 [39,40]	Sweden	RCT, 152 (stress-subgroup of total 211), Three arms: CBT (52)/ RTW-I (49)/ COMBO (51)	42.7 (9.7), 88 %	Adjustment disorder and Exhaustion disorder	Adjustment disorder (DSM-IV) and (Swedish ICD-10: F43.8)	CBT, RTW-I, combination; Individual, 10-25 weeks, CBT 10/ RTW-I 10/COMBO 10- 25, weekly sessions (NS)	Licensed psychologists, with one to eight years of experience of working with CBT	12 months	**Primary:** CSR, Sick-leave days **Secondary:** HADS, MADRS-S, PSS, SMBQ (Exhaustion-patients only)	Pre-post: CSR CBT vs RTW-I ($p < .01$), not sustained at 12MFU Pre-post: SMBQ CBT vs RTW-I $p < .05$, not sustained at 12MFU No significant between-differences in sick-leave.	Pre-post: CSR CBT vs RTW-I d = 0.52<br><br>Pre-post: SMBQ CBT vs RTW-I d = 0.38	Within pre-12MFU ($p < .05$) all secondary outcomes for all treatments ($0.98 \leq d \leq 2.72$)
Sandahl et al., 2011 [41]	Sweden	RCT, 117 (11), Three arms: Focused psychodynamic (37)/ Cognitive (40)/ WLC (40)	43.0 (1.8), 70 %	Work-related depression and burnout	Work-related MDD, dysthymia, or maladaptive stress reaction (DSM-IV)	FGT/CGT, Group, 6 months, 18/14 (90-120 min)	Licensed psychologist and psychiatrist	12 months	Primary not defined. CPRS-S-A, SCL-90, OLBI	No significant outcomes.	NS	Significant within improvements pre-12MFU in all groups on all measures ($0.5 \leq d \leq 1.7$)
Schene et al., 2007 [42]	The Netherlands	RCT, 62 (3), TAU (32)	45.9 (7.4), 52 %	Work-related depression	MDD (DSM-IV).	Occupational therapy, individual+group, 6 months, 36 (60-120 min)	Three supervised senior psychiatric residents	12 and 42 months	**Primary:** BDI and work resumption **Secondary:** QOS and economic evaluation	No significant outcomes.	NS	NS

Stenlund et al., 2009 [43,44]	Sweden	RCT, 136 (29), Cognitive Behavioral rehabilitation (67)/ Qigong (69)	41.6 (7.5), 71 %	Burnout, exhaustion disorder	44% (F30-F39), 2% (F40-42) 54% (F43)	Cognitive Behavioral rehabilitation, Qigong, Group, 12 months; CBR, 30 (3h), Qigong, 56 (1 h)	Physiotherapist trained in Qigong, leader "specially trained in CBR"	36 months	**Primary:** SMBQ, **Secondary:** ELSS, CIS, CPRS-S-A, Sick Leave Rates.	Pre-post: No significant effects	NS	Between SMBQ 36MFU ($p = .035$) in favor of CBR.<br><br>Between pre-36MFU decrease in medication for depression ($p = .01$) in CBR
Ugwoke et al., 2018 [45]	Nigeria	RCT, 54 (0), WLC (26)	36.7 (5.3), 56 %	Burnout	No	REBT, Group, 12 weeks, 12 (2 h)	Authors. Which of the authors, or level of education not described.	Yes, though NS when	Primary not defined. TBS, PED	Pre-post, time x group: PED ($p < .001$), TBS ($p < .001$) at post and follow-up	$\eta^2p = .9 – 1.0$	NS
van der Klink et al., 2003 [46]	The Netherlands	Prospective cluster-randomized controlled trial, 192 (75), CAU (83),	40.3 (8.5), 37 %	Adjustment disorder	Adjustment disorder (AD)	Occupational intervention, Individual, 12 weeks, 8-9 (90 min)	Occupational physician	9 months	Primary not defined. 4DSQ, SCL-90, Mastery Scale, Absenteeism	Pre-post: Lower absenteeism in intervention ($p < .001$). Time to return to work, time to full return to work, and duration of sickness leave were significantly shorter for the intervention group ($p < 0.05$).	NS	At 9MFU all participants had returned to work.
van Noppen et al., 2020 [47]	Belgium	RCT, 15 (0), Sham-treatment of tDCS (7)	44.8 (5.8), 67 %	Burnout	No	Behavioral Therapy + tDCS, Individual, 3 weeks, 3 + daily atDCS (NS)	Psychologist	No	Primary not defined. MBS, BDI, QoL, RBANS (Attention index, Working memory Encoding, Retrieval)	RBANS Attention index: interaction tDCS x time ($p < .05$)	NS	Within pre-post BDI ($p = .01$) and MBS ($p = .01$)
Verkuil et al., 2011 [48]	The Netherlands	RCT, 62 (0), Three arms: Worry postponement and disengagement (22) / worry registration (15) / TAU (25)	NS	Work stress.	AD, unspecified somatoform disorder (burnout) or severe work problems (axis IV; DSM-IV).	WPD intervention prior to a standard stress management group therapy, Individual (self-help), 2 weeks, 0	NS	3 months	Primary not defined. SHC, STAI-T, BDI-II	Pre-3MFU: TAU vs WPD Decreases in somatoform symptoms ($p = .017$), anxiety ($p = .048$)	NS	NS
Willert et al., 2009 [49,50]	Denmark	RCT, 102 (14), WLC	45.0 (NS), 82 %	Work-related stress	No	CBT, Group, 12 weeks, 8 (180 min)	Clinical psychologists	3 months	Primary not defined. PSS-10, Brief COPE questionnaire, self-rated absenteeism, sick-leave data (public transfer database)	Pre-post: PSS-10 ($p < .001$) Positive reframing ($p = .01$)	Pre-post: PSS-10 $d = 0.92$ Positive reframing $d = 0.49$	Between pre-post lower self-rated absenteeism ($p < .05$)<br><br>No significant effects on sick-leave data.

Zielhorst et al., 2015 [51]	The Netherlands	2x2 Pretest-posttest mixed design, 101 (16), Four arms: Game + Therapy (17) / Therapy only (20) / Game only (27) / No intervention (21)	40.0 (9.1), 49 %	Burnout Syndrome	No	Digital game + CBT, Internet, NS, NS	Therapists, not furtherly specified	No	Primary not defined. DASS (Burnout symptoms, Self-esteem, Coping skills), OLBI, SISE	Pre post: DASS ($p < .00$) in favor of Game + therapy and Therapy	Pre-post: $\eta^2 p$ = .210	NS
**Within studies**												
Adina et al., 2021[52]	Romania	One-group pre-and posttest design, 30 (NS), No	40.2 (7.2), 93 %	Burnout syndrome	Burnout syndrome (ICD-11)	CBT+hypnotherapy, Individual, 8-10 weeks, 8-10 (60 min)	NS	No	Primary not defined. MBI, DECAS, PID-5, SWS	Pre-post: Significant effects ($p < .05$) on all dimensions of MBI, Extraversion and Emotional stability on DECAS, all traits in PID-5 except for Irresponsibility and Risk assumption, all Type A-behaviors in SWS	NS	NS
Ekstedt et al., 2009 [53]	Sweden	Open clinical trial, 39 (1), healthy control (16)	43.6 (2.0), 72 %	Burnout; Fatigue due to work- related stress	ED (Swedish ICD-10: F43.8)	CBT-based MMI, Group and individual, 6 months, 30-40 (NS).	Multimodal team, not furtherly specified	No	**Primary:** Fatigue index, sleep quality index **Secondary:** SMBQ, BDI, BAI, RTW	Pre-post: Significant effects ($p < .01$) on all outcomes.	NS	Recovery from fatigue was related to a reduction of the arousal from sleep and was the best predictor of return to work.
Ekvall Hansson et al, 2009 [54]	Sweden	Retrospective study, 13 (0), No	Mdn = 40, 77 %	Burnout	No	CBT-based MMI, Individual, 18 weeks, 18 (45 min).	Occupational therapist and physio-therapist	3 and 6 months from baseline	Primary not defined. SMBQ, EQ5D VAS, sick leave (yes/no)	Pre-6MFU: SMBQ (p = .001) EQ5D VAS (p = .001)	NS	NS
Firth-Cozens & Hardy, 1992 [55]	England	Longitudinal, 90 (0), No	NS, 46 %	Job stress	Clinical depression (according to BDI)	Eclectic, Individual, NS, NS	Clinical psychologists	No	Primary not defined. AWI	Pre-post: AWI subscales improved ($p < .05$), but one (perceived variety in the job)	NS	Changes in perception of work and clinical changes were correlated.
Grigorescu et al., 2020 [56]	Romania	Experimental (pilot), 23 (0), No intervention (12)	41.7 (5.6), NS	Burnout syndrome	No	Psychodrama, Group, 10 weeks, 10 (2,5 h)	NS	No	Primary not defined. CBI-R, CAS, SDS, CERQ	For experimental group: Depression (p = .05)	NS	NS
Kjellgren et al., 2011 [57]	Sweden	Pilot study, 6 (0), No	42.7 (9.3), 67 %	Burnout syndrome	No	Flotation tank together with (unspecified) psychotherapy, Individual, 10 weeks, 30 (45-105 min)	Psychologist	No	Primary not defined. HADS, GHQ-12, PAI, SQ, VAS Worst pain	Pre-post: Depression ($p < .05$) Anxiety ($p < .05$) GHQ-12 ($p < .05$) VAS ($p < .05$) PAI ($p < .05$) SQ ($p < .05$)	Depression r = .58 Anxiety r = .65 GHQ-12 r = .63 VAS r = .63 PAI r = .57 SQ r = .53	NS

Lindström et al., 2016 [58]	Sweden	Single group design with repeated measures, 16 (0), No	43.0 (3.3), 81 %	Clinical burnout	No	CBT, system theory; Group, 12 weeks, 8 (NS)	Author with experience of counselling parents.	6 months	Primary not defined. SMBQ, PBSE	Pre-6MFU: SMBQ (p = .01), PBSE (p = .04)	NS	NS
Meesters et al., 2012 [59]	The Netherlands	Natural field study, 170 (21), 99 participants in follow-up, No	43.0 (9.0), 51 %	Burnout	No	CBT, Group, 13 weeks, 9 (one day à 8 h)	Experienced clinical psychologist	12 months	Primary not defined. UBOS, hours spent on work, SFQ, BDI, UCL, SCL-90-R	Pre-12MFU: Significant outcomes on all measures (.00 < p < .04)	Pre-12MFU: All measures ES = 0.3 – 1.29	NS
Millet, 2008 [60]	Sweden	Pilot study, 32 (0), No	NS	Exhaustion syndrome	No (but refers to Swedish definition of ED)	Vocational rehabilitation (garden-based), Individual and group, 21-29 weeks, minimum of 3-4 hours, 5 times a week recommended	Rehabilitation workers, not furtherly specified	2 months	Primary not defined. SF-36, levels of salivary cortisol, SE-scale, KSQ; abbreviated versions of LCS and SPS	Pre-post: SE-scale (stress) (p < .001) KSQ (p < .001)	NS	Significantly less salivary cortisol at the end of treatment (p = 0.025) and at 2MFU (p < .001) compared to 2 weeks before start.
Mommersteeg et al., 2006 [61]	The Netherlands	Pilot study, 43 (3), Healthy control (21)	45.0 (8.0), 68 %	Burnout	Work-related neurasthenia (clinical burnout), ICD-10	CBT, NS, on average 6 months, 14 (NS)	NS	No	Primary not defined. MBI-GS, CIS-20R, CES-D, GSKS, SCL-90	Within pre-post: MBI-GS (p < .001), CIS-20R (p < 001), CES-D (p < .001), GSKS (p < .001) SCL-90 (p < .01)	NS	The burnout group had significantly lower cortisol levels after awakening (p<.001, η^2p = .28). No difference in cortisol level during the day.
Mommersteeg et al., 2006 [62]	The Netherlands	Longitudinal study, 74 (21), No	43.9 (8.7), 28 %	Burnout	Work-related neurasthenia (clinical burnout) in ICD	CBT, individual, min 3 months, max 20 (NS)	NS	6 months	Primary not defined. MBI-GS, SLC-90	Within pre-post: MBI-GS, SLC-90 (p < .001) No parametric tests for 6MFU	NS	No changes in cortisol levels.
Orosz et al., 2021 [63]	Switzerland	Naturalistic Single-group study, 71 (3), No	46.8 (9.9), 45 %	Burnout	Major depression (ICD F32/F33)	Multimodal program, Individual and group (therapy, massage and training), 6 weeks. 3 individual CBT-sessions, 19 h group total	Experienced therapists	No	Primary not defined. MBI, BDI, ISI<br><br>Brain-derived neurotrophic factor, heart rate variability and sleep time	Pre-post: MBI (p < .001) BDI (p < .001) ISI (p < .001)	NS	Significant increase in serum BDNF (p < .001).
Pálsdóttir et al., 2013 [64]	Sweden	Clinical trial, 21 (6), No	47.0 (NS), 91 %	Stress-related mental illnesses	Adjustment disorder and reaction to severe stress (ICD F43); depression (ICD F32.0, ICD F32.1)	Nature-based vocational rehabilitation, Group and individual, 12 weeks, 192 (NS)	Multiprofessional team	3 and 12 months	Primary not defined. Oval-pd, OSA-F, EQ-VAS, SCI-93, SOC-13	Pre-3MFU-12MFU: Oval-pd, OSA-F, EQ-VAS, SCI-93, SOC-13 (p < .05 for all measures)	NS	NS
Sahlin et al., 2014 [65]	Sweden	Observational follow-up study, 44 (4), No	NS in terms of M (SD), 100 %	Individuals with increasing stress-related problems	No	Nature-based MMI, Group, 12 weeks, 24 (3h)	Team of: psychotherapist, physiotherapist, gardener, biologist, occupational therapist	6 and 12 months	**Primary:** SMBQ, WAI, **Secondary:** Sick leave (WAI item 5), KSQ	Within pre-post: SMBQ not significant Pre-6MFU and pre-12MFU: SMBQ (significant regarding CI)	NS	WAI5 only significant pre-12MFU<br><br>No significant effects of KSQ

Sahlin et al., 2015 [66]	Sweden	Observational follow-up study, 117 (15), Occupational Health Service Group (45)	45.0 (NS), 88 %	Stress-related mental disorders	Work-related ICD-diagnoses in the F43 category and/or depression (F32), and/or anxiety (F41)	Nature-based MMR, Group, Phase 1: 16 weeks, 64 (3h); Phase 2: 12 weeks with gradual return to work or study with corresponding decrease in participation in treatment	Team of: psychotherapist, physiotherapist, gardener, biologist, occupational therapist	6 and 12 months	Primary not defined. SMBQ (%, cutoff = 4.4), BDI, BAI, PGWB, Return-to-work, Sick leave Health Care Utilization (data from the Swedish database VEGA)	Pre-12MFU: WAI (significant regarding CI) Pre-post-6MFU-12MFU: Significant difference in proportions SMBQ Pre-post-6MFU-12MFU on BDI (p < .001) Pre-post-6MFU-12MFU on BAI (p < .001)	SMBQ pre-post: 23.5 % difference [95 % CI, 8.6; 36.9] SMBQ pre-6MFU: 23.9 % difference [95 % CI, 7.9; 38.2] SMBQ pre-12MFU: 40 % difference [95 % CI, 19.6; 55.8]	Significant within pre-post-6MFU-12MFU on PGWB (p < .001)<br><br>Significant within pre-post-6MFU-12MFU on sick-leave (p < .001)
Steensma et al., 2007 [67]	The Netherlands	One-group-pretest-posttest design, 20, No	NS in terms of M (SD), 75 %	Protracted illness due to stress or burnout	No	Resilience program, Group, 26 weeks, NS	NS	No	Primary not defined. UCL, BDI	Pre-post: BDI (p < .000) Growth in resilience linked to reduction in depression (p < .01).	NS	Active coping styles used significantly more.
Thomas et al., 2020 [68]	USA	Pilot study, 166 (NS), No	46.0 (NS), 73 %	Work stress, burnout	Depression, anxiety, AD, occupational stress disorder.	WRG, Group and individual, no set length, twice/week, on average 4 sessions (90 min)	Clinical psychologist	No	Primary not defined. Return to work, AOQ	85 % returned to work with no days off during the study period.	NS	Involvement in the WRG also led to improvement in self-reported symptom severity, with a decrease in the average AOQ score of 4.5 points.
van de Leur et al., 2020 [69]	Sweden	Open clinical trial, 390 (11), No	43.7 (9.4), 88 %	Stress-induced Exhaustion Disorder (SED)	ED (Swedish ICD-10: F 43.8)	CBT-based MMI, Group and individual, 24 weeks, 35 (1-2 h)	Licensed psychologists and physiotherapists, MDs	12 months	**Primary:** KEDS, RTW **Secondary:** HADS, EQ5D, ISI, SMBQ	Pre-12MFU: KEDS (p < . 01) HADS depression (p < . 01) HADS anxiety (p < . 01) SMBQ (p < . 01) ISI (p < . 01) EQ5D (p < . 01)	Pre-12MFU: KEDS d = 1.7 HADS depression d = 1.2 HADS anxiety d = 1.2 SMBQ d = 1.8 ISI d = 1.1 EQ5D d = 0.9	Within pre-follow-up 50 % increase in RTW
**Other**												
Hätinen et al., 2009 [70]	Finland	Open clinical trial, 85 (16), No	NS	Burnout	No (Z73.0 in ICD (Burnout)	Traditional and participatory intervention (generic coping), Group and individual, 12+5 days, NS	MDs, licensed psychologist and licensed physiotherapist	No	Primary not defined. MBI-GS (exhaustion, cynicism and reduced professional efficacy), *Time pressure, Job control, Workplace-climate,* BDI, JDS (Job satisfaction)	Significant difference in three trajectories of burnout-patients: High burnout benefited, high burnout- not benefited. Low burnout	NS	Significant difference in three trajectories of burnout-patients: High burnout benefited, high burnout- not benefited, low burnout

Kinnunen et al., 2019 [71]	Finland	RCT, 218 (29), TAU (109)	47.8 (7.8), 80 %	Burnout	No	ACT, Group-/web-based, 8 weeks, 8 (NS)	Two psychologists.	4 months	Primary not defined. BBI, FFMQ, Practices of mindfulness	Six profiles showed different baseline levels and change patterns for both burnout and mindfulness skills.	NS	There were differences in the levels of changes between the profiles. Profiles 1 and 5 benefited greatly from the intervention
van der Meulen et al., 2021 [72]	The Netherlands	Mediation analysis of samples in two open clinical trials, 124 (NS), No	43.9 (10.2), 77 %	Burnout related complaints and stress	No	Mindful2work, Group, 6 weeks, 6 (2h)	NS	6 weeks	Primary not defined. PSS, CIS, FFMQ-SF, SCS-SF	Pre-post changes in mindfulness (FFMQ-SF) mediated the effects on perceived stress (PSS) and chronic fatigue (CIS).	NS	Pre-post: increases in mindfulness (FFMQ-SF) led to more self-compassion (CIS), which in turn led to less stress (PSS)

NS = Not specified. Mdn = Median. MFU= Months follow-up. RCT = Randomized controlled trial. YFU = Years follow-up. ***Diagnosis:*** AD = Adjustment disorder. ED = Exhaustion Disorder. MDD = Major Depressive Disorder. PTSD = Post Traumatic Stress Disorder. ***Outcome:*** 4DSQ = Four-Dimensional Symptom Questionnaire. AAQ-2 = Acceptance and Action Questionnaire. AOQ = Adult Outcomes Questionnaire. AUDIT = Alcohol Use Disorders Identification Test. AWI = Aspects of Work Inventory. BAI = Beck Anxiety Inventory. BADS = Behavioral Activation for Depression Scale. BBI = Bergen Burnout Indicator. BDI = Beck Depression Inventory. BNSQ = Basic Nordic Sleep Questionnaire. CAS = Catell Anxiety Scale. CBI-R = Cambridge Behavioural Inventory Revised. CERQ = Cognitive-Emotion Regulation Questionnaire. CES-D = Center for Epidemiologic Studies Depression Scale. CFQ = Cognitive Failure Questionnaire. CIS = Checklist Individual Strength. Brief-COPE = Coping Orientation to Problems Experienced Inventory. CPRS-S-A = Comprehensive Psychopathological Rating Scale-Self-Affective. CSR = Clinician Severity Rating. DASS = Depression Anxiety Stress Scales. DSP = Derogatis Stress Profile. ELSS = Everyday Life Stress Scale. EQ-VAS = EuroQol – visual analogue scales. EQ5D = EuroQol five dimensions scale. ERI = Effort reward imbalance. EROS = Environmental Reward Observation Scale. FFMQ = Five Facets Mindfulness Questionnaire. GAD-7 = Generalized Anxiety Disorder 7-item Scale. GHQ-30 = General Health Questionnaire. GSKS = Groninger Slaap Kwaliteits Schaal (Groningen Sleep Quality Scale). HADS = Hospital Anxiety and Depression Scale. IDAS = Inventory of Depression and Anxiety Symptoms. ISI = Insomnia Severity Index. JDS = Job Diagnostic Survey. KEDS = Karolinska Exhaustion Disorder Scale. KIMS-D = Kentucky Inventory of Mindfulness Skills. KSQ = Karolinska Sleep Questionnaire. LCS = Rotter Locus of Control Scale. MADRS-S = Montgomery Åsberg Depression Rating Scale. MANSA = Manchester Short Assessment of Quality of Life. MBI-GS = Maslach Burnout Inventory - General Survey. MBS = Maslach Burnout Scale. MDI = Major Depression Inventory. OLBI-S = Oldenburg Burnout Inventory. OSA = Occupational Self-Assessment. OSA-F = Occupational Self-Assessment-Function. OSI = Occupational Stress Inventory. Oval-pd = Occupational Value. PAI = Pain Area Inventory. PANAS = Positive and Negative Affect Schedule. PBSE = Performance-based self-esteem scale. PED = Profile of Emotional Distress. PGWB = Psychological General Well-Being Index. PID-5 = Personality Inventory for DSM-5. PM scale = Pearlin Mastery Scale. POMS-37 = Profile of Moods States. PPA = Personal Project Analysis inventory. PSE = Present State Examination. PSS = Perceived Stress Scale. QoL = Quality of Life. QOS = Questionnaire Organization Stress. RBANS = Repeatable Battery for the Assessment of Neuropsychological Status. RTW = Return to work. SA = Sick Absenteeism. SBI = School burnout questionnaire. SCI-93 = Stress and Crises Inventory. SCL-90R = Symtoms checklist-90-Revised. SCS-SF = Self-Compassion Scale - Short Form. SCSI = Structured Clinical Stress Interview. SDS = Zung Self-Rating Depression Scale. SE-scale = Stress-Energi (Stress-Energy scale). SF-36 = Swedish Health Survey. SFQ = Shortened Fatigue Questionnaire. SHC = Subjective Health Complaints Questionnaire. SISE = Single Item Self Esteem scale. SMBQ = Shirom Melamed Burnout Questionnaire. SoC = Sense of coherence scale. SPS = Social Provisions Scale. SQ = Sleep Quality. STAI-T = State Trait Anxiety Inventory – trait version. SWED-QUAL = Swedish Health-Related Quality of Life Survey. SWLS = Satisfaction with Life Scale. SWS = Survey of Work Style. TBS = Teacher Burnout Scale. TiC-P = Trimbos and Institute of Medical Technology Assessment Cost Questionnaire for Psychiatry. TSM = Teacher Stress Measure. UBOS = Utrecht Burnout Scale. UCL = Utrecht coping list. VAS = Visual Analog Scale. WAI = Work Ability Index. WEMS = Work Experience Measurement Scale. WHO-5 = World Health Organization- Five Well-Being Index. WSAS = Work and Social Adjustment Scale. **Treatment/control:** ACT = Acceptance and Commitment Therapy. atDCS = Anodal transcranial direct current stimulation. CAU = Care as usual. CBT = Cognitive Behavioral Therapy. COMBO = Combination of RTW-I and CBT. MBCT = Mindfulness-Based Cognitive Therapy. MMI = Multimodal intervention. MMR = Multimodal Rehabilitation. RTW-I = Return to work intervention. SMT = Stress management training. TAU = Treatment as usual. tDCS = Transcranial direct current stimulation. WDI = Workplace dialogue intervention. WLC = Wait-list control. WPD = Worry postponement and disengagement. WRG = Work recovery group.

1 Eklund, 2013, 2017; Eklund & Erlandsson, 2013; Wästberg et al., 2016

2 Eskilsson et al., 2017; Gavelin et al., 2017; Malmberg Gavelin et al., 2018

References

1. Anclair M, Hiltunen AJ. Cognitive Behavioral Therapy for Stress-Related Problems. Clin Case Stud [Internet]. 2014 Dec 12;13(6):472–86. Available from: http://journals.sagepub.com/doi/10.1177/1534650114522090
2. Barkham M, Shapiro DA. Brief psychotherapeutic interventions for job-related distress: A Pilot study of Prescriptive and Exploratory therapy. Couns Psychol Q [Internet]. 1990 Apr 27;3(2):133–47. Available from: http://www.tandfonline.com/doi/full/10.1080/09515079008254242
3. Gyllensten K, Palmer S. Working with a Client Suffering From Workplace Stress in a Primary Care Setting: A Cognitive Behavioural Case Study [Internet]. 2005. Available from: https://www.researchgate.net/publication/335987906
4. Beck BD, Hansen ÅM, Gold C. Coping with Work-Related Stress through Guided Imagery and Music (GIM): Randomized Controlled Trial. J Music Ther [Internet]. 2015;52(3):323–52. Available from: https://academic.oup.com/jmt/article-lookup/doi/10.1093/jmt/thv011
5. Bertoch MR, Nielsen EC, Curley JR, Borg WR. Reducing Teacher Stress. The Journal of Experimental Education [Internet]. 1989 Jan 16;57(2):117–28. Available from: http://www.tandfonline.com/doi/abs/10.1080/00220973.1989.10806500
6. Beutel ME, Knickenberg RJ, Krug B, Mund S, Schattenburg L, Zwerenz R. Psychodynamic Focal Group Treatment for Psychosomatic Inpatients–with an Emphasis on Work-Related Conflicts. Int J Group Psychother [Internet]. 2006 Jul 21;56(3):285–306. Available from: https://www.tandfonline.com/doi/full/10.1521/ijgp.2006.56.3.285
7. Buxton AE, Remmers C, Unger HP, Plinz N, Michalak J. Treating Depression Mindfully in a Day Hospital: a Randomised Controlled Pilot Study. Mindfulness (N Y) [Internet]. 2020 Feb 28;11(2):384–400. Available from: http://link.springer.com/10.1007/s12671-019-01233-4
8. Dalgaard VL, Andersen LPS, Andersen JH, Willert MV, Carstensen O, Glasscock DJ. Work-focused cognitive behavioral intervention for psychological complaints in patients on sick leave due to work-related stress: Results from a randomized controlled trial. J Negat Results Biomed [Internet]. 2017 Dec 22;16(1):13. Available from: http://jnrbm.biomedcentral.com/articles/10.1186/s12952-017-0078-z
9. de Vente W, Kamphuis JH, Emmelkamp PMG, Blonk RWB. Individual and group cognitive-behavioral treatment for work-related stress complaints and sickness absence: A randomized controlled trial. J Occup Health Psychol [Internet]. 2008 Jul;13(3):214–31. Available from: http://doi.apa.org/getdoi.cfm?doi=10.1037/1076-8998.13.3.214
10. Eklund M, Erlandsson LK. Return to Work Outcomes of the Redesigning Daily Occupations (ReDO) Program for Women with Stress-Related Disorders—A Comparative Study. Women Health [Internet]. 2011 Nov;51(7):676–92. Available from: http://www.tandfonline.com/doi/abs/10.1080/03630242.2011.618215
11. Eklund M, Erlandsson LK. Quality of life and client satisfaction as outcomes of the Redesigning Daily Occupations (ReDO) programme for women with stress-related disorders: A comparative study. Work [Internet]. 2013 Sep 27;46(1):51–8. Available from: https://www.medra.org/servlet/aliasResolver?alias=iospress&doi=10.3233/WOR-121524

12. Wästberg BA, Erlandsson LK, Eklund M. Women's perceived work environment after stress-related rehabilitation: experiences from the ReDO project. Disabil Rehabil [Internet]. 2016 Mar 12;38(6):528–34. Available from: http://www.tandfonline.com/doi/full/10.3109/09638288.2015.1046567
13. Eklund M. Anxiety, Depression, and Stress Among Women in Work Rehabilitation for Stress-Related Disorders. Int J Ment Health [Internet]. 2013 Dec 10;42(4):34–47. Available from: https://www.tandfonline.com/doi/full/10.2753/IMH0020-7411420402
14. Eklund M. Minor long-term effects 3-4 years after the ReDO™ intervention for women with stress-related disorders: A focus on sick leave rate, everyday occupations and well-being. Work [Internet]. 2017 Dec 13;58(4):527–36. Available from: https://www.medra.org/servlet/aliasResolver?alias=iospress&doi=10.3233/WOR-172639
15. Ezenwaji IO, Eseadi C, Ugwoke SC, Vita-Agundu UC, Edikpa E, Okeke FC, et al. A group-focused rational emotive behavior coaching for management of academic burnout among undergraduate students. Medicine [Internet]. 2019 Jul 1;98(30):e16352. Available from: https://journals.lww.com/00005792-201907260-00010
16. Ezeudu FO, Nwoji IHN, Dave-Ugwu PO, Abaeme DO, Ikegbunna NR, Agugu CV, et al. Intervention for burnout among chemistry education undergraduates in Nigeria. Journal of International Medical Research [Internet]. 2020 Jan 9;48(1):030006051986783. Available from: http://journals.sagepub.com/doi/10.1177/0300060519867832
17. Fang CM, McMahon K, Miller ML, Rosenthal MZ. A pilot study investigating the efficacy of brief, phone-based, behavioral interventions for burnout in graduate students. J Clin Psychol [Internet]. 2021 Dec 13;77(12):2725–45. Available from: https://onlinelibrary.wiley.com/doi/10.1002/jclp.23245
18. Finnes A, Enebrink P, Sampaio F, Sorjonen K, Dahl J, Ghaderi A, et al. Cost-Effectiveness of Acceptance and Commitment Therapy and a Workplace Intervention for Employees on Sickness Absence due to Mental Disorders. J Occup Environ Med [Internet]. 2017 Dec;59(12):1211–20. Available from: https://journals.lww.com/00043764-201712000-00012
19. Finnes A, Hoch JS, Enebrink P, Dahl J, Ghaderi A, Nager A, et al. Economic evaluation of return-to-work interventions for mental disorder-related sickness absence: two years follow-up of a randomized clinical trial. Scand J Work Environ Health [Internet]. 2022 May 1;48(4):264–72. Available from: http://www.sjweh.fi/show_abstract.php?abstract_id=4012
20. Firth J, Shapiro DA. An evaluation of psychotherapy for job-related distress. Journal of Occupational Psychology [Internet]. 1986 Jun;59(2):111–9. Available from: https://onlinelibrary.wiley.com/doi/10.1111/j.2044-8325.1986.tb00218.x
21. Glasscock DJ, Carstensen O, Dalgaard VL. Recovery from work-related stress: a randomized controlled trial of a stress management intervention in a clinical sample. Int Arch Occup Environ Health [Internet]. 2018 Aug 28;91(6):675–87. Available from: http://link.springer.com/10.1007/s00420-018-1314-7
22. Grahn P, Pálsdóttir AM, Ottosson J, Jonsdottir IH. Longer Nature-Based Rehabilitation May Contribute to a Faster Return to Work in Patients with Reactions to Severe Stress and/or Depression. Int J Environ Res Public Health [Internet]. 2017 Oct 27;14(11):1310. Available from: http://www.mdpi.com/1660-4601/14/11/1310

23. Grensman A, Acharya BD, Wändell P, Nilsson GH, Falkenberg T, Sundin Ö, et al. Effect of traditional yoga, mindfulness–based cognitive therapy, and cognitive behavioral therapy, on health related quality of life: a randomized controlled trial on patients on sick leave because of burnout. BMC Complement Altern Med [Internet]. 2018 Dec 6;18(1):80. Available from: https://bmccomplementalternmed.biomedcentral.com/articles/10.1186/s12906-018-2141-9
24. Hätinen M, Kinnunen U, Pekkonen M, Kalimo R. Comparing two burnout interventions: Perceived job control mediates decreases in burnout. Int J Stress Manag [Internet]. 2007 Aug;14(3):227–48. Available from: http://doi.apa.org/getdoi.cfm?doi=10.1037/1072-5245.14.3.227
25. Igbokwe UL, Nwokenna EN, Eseadi C, Ogbonna CS, Nnadi EM, Ololo KO, et al. Intervention for burnout among English education undergraduates: implications for curriculum innovation. Medicine [Internet]. 2019 Jun 1;98(26):e16219. Available from: https://journals.lww.com/00005792-201906280-00088
26. Lappalainen P, Kaipainen K, Lappalainen R, Hoffrén H, Myllymäki T, Kinnunen ML, et al. Feasibility of a Personal Health Technology-Based Psychological Intervention for Men with Stress and Mood Problems: Randomized Controlled Pilot Trial. JMIR Res Protoc [Internet]. 2013 Jan 9;2(1):e1. Available from: http://www.researchprotocols.org/2013/1/e1/
27. Lindsäter E, Axelsson E, Salomonsson S, Santoft F, Ejeby K, Ljótsson B, et al. Internet-Based Cognitive Behavioral Therapy for Chronic Stress: A Randomized Controlled Trial. Psychother Psychosom [Internet]. 2018;87(5):296–305. Available from: https://www.karger.com/Article/FullText/490742
28. Lindsäter E, Axelsson E, Salomonsson S, Santoft F, Ljótsson B, Åkerstedt T, et al. The mediating role of insomnia severity in internet-based cognitive behavioral therapy for chronic stress: Secondary analysis of a randomized controlled trial. Behaviour Research and Therapy [Internet]. 2021 Jan;136(April 2020):103782. Available from: https://linkinghub.elsevier.com/retrieve/pii/S0005796720302369
29. Gavelin HM, Boraxbekk CJ, Stenlund T, Järvholm LS, Neely AS. Effects of a process-based cognitive training intervention for patients with stress-related exhaustion. Stress [Internet]. 2015 Sep 3;18(5):578–88. Available from: http://www.tandfonline.com/doi/full/10.3109/10253890.2015.1064892
30. Eskilsson T, Slunga Järvholm L, Malmberg Gavelin H, Stigsdotter Neely A, Boraxbekk CJ. Aerobic training for improved memory in patients with stress-related exhaustion: a randomized controlled trial. BMC Psychiatry [Internet]. 2017 Dec 2;17(1):322. Available from: https://bmcpsychiatry.biomedcentral.com/articles/10.1186/s12888-017-1457-1
31. Gavelin HM, Neely AS, Andersson M, Eskilsson T, Järvholm LS, Boraxbekk CJ. Neural activation in stress-related exhaustion: Cross-sectional observations and interventional effects. Psychiatry Res Neuroimaging [Internet]. 2017 Nov;269:17–25. Available from: https://linkinghub.elsevier.com/retrieve/pii/S0925492717300549
32. Malmberg Gavelin H, Eskilsson T, Boraxbekk CJ, Josefsson M, Stigsdotter Neely A, Slunga Järvholm L. Rehabilitation for improved cognition in patients with stress-related exhaustion disorder: RECO – a randomized clinical trial. Stress [Internet]. 2018 Jul 4;21(4):279–91. Available from: https://www.tandfonline.com/doi/full/10.1080/10253890.2018.1461833

33. Netterstrøm B, Bech P. Effect of a multidisciplinary stress treatment programme on the return to work rate for persons with work-related stress. A non-randomized controlled study from a stress clinic. BMC Public Health [Internet]. 2010 Dec 1;10(1):658. Available from: https://bmcpublichealth.biomedcentral.com/articles/10.1186/1471-2458-10-658
34. Oloidi FJ, Sewagegn AA, Amanambu OV, Umeano BC, Ilechukwu LC, Palmieri F. Academic burnout among undergraduate history students: Effect of an intervention. Medicine [Internet]. 2022;101(7):e28886–e28886. Available from: https://search.ebscohost.com/login.aspx?direct=true&db=cin20&AN=155473309&site=ehost-live
35. Oosterholt BG, van der Linden D, Maes JH, Verbraak MJ, Kompier MA. Burned out cognition – cognitive functioning of burnout patients before and after a period with psychological treatment. Scand J Work Environ Health [Internet]. 2012 Jul;38(4):358–69. Available from: http://www.sjweh.fi/show_abstract.php?abstract_id=3256
36. Oosterholt BG, Maes JHR, van der Linden D, Verbraak MJPM, Kompier MAJ. Getting better, but not well: A 1.5 year follow-up of cognitive performance and cortisol levels in clinical and non-Clinical burnout. Biol Psychol [Internet]. 2016 May 1;117:89–99. Available from: https://linkinghub.elsevier.com/retrieve/pii/S0301051116300461
37. Persson Asplund R, Dagöö J, Fjellström I, Niemi L, Hansson K, Zeraati F, et al. Internet-based stress management for distressed managers: results from a randomised controlled trial. Occup Environ Med [Internet]. 2018 Feb;75(2):105–13. Available from: https://oem.bmj.com/lookup/doi/10.1136/oemed-2017-104458
38. Salmela-aro K, Näätänen P, Nurmi J erik. The role of work-related personal projects during two burnout interventions: a longitudinal study. Work Stress [Internet]. 2004 Jul;18(3):208–30. Available from: http://www.tandfonline.com/doi/abs/10.1080/02678370412331317480
39. Salomonsson S, Santoft F, Lindsäter E, Ejeby K, Ingvar M, Ljótsson B, et al. Effects of cognitive behavioural therapy and return-to-work intervention for patients on sick leave due to stress-related disorders: Results from a randomized trial. Scand J Psychol [Internet]. 2020 Apr 6;61(2):281–9. Available from: https://onlinelibrary.wiley.com/doi/10.1111/sjop.12590
40. Santoft F, Salomonsson S, Hesser H, Lindsäter E, Ljótsson B, Lekander M, et al. Mediators of Change in Cognitive Behavior Therapy for Clinical Burnout. Behav Ther [Internet]. 2019 May;50(3):475–88. Available from: https://linkinghub.elsevier.com/retrieve/pii/S0005789418301084
41. Sandahl C, Lundberg U, Lindgren A, Rylander G, Herlofson J, Nygren Å, et al. Two Forms of Group Therapy and Individual Treatment of Work-Related Depression: A One-Year Follow-Up Study. Int J Group Psychother [Internet]. 2011 Oct 25;61(4):538–55. Available from: https://www.tandfonline.com/doi/full/10.1521/ijgp.2011.61.4.538
42. Schene AH, Koeter MWJ, Kikkert MJ, Swinkels JA, McCrone P. Adjuvant occupational therapy for work-related major depression works: randomized trial including economic evaluation. Psychol Med [Internet]. 2007 Mar 20;37(03):351. Available from: http://www.journals.cambridge.org/abstract_S0033291706009366

43. Stenlund T, Ahlgren C, Lindahl B, Burell G, Steinholtz K, Edlund C, et al. Cognitively Oriented Behavioral Rehabilitation in Combination with Qigong for Patients on Long-Term Sick Leave Because of Burnout: REST—A Randomized Clinical Trial. Int J Behav Med [Internet]. 2009 Sep 16;16(3):294–303. Available from: http://link.springer.com/10.1007/s12529-008-9011-7
44. Stenlund T, Nordin M, Järvholm L. Effects of rehabilitation programmes for patients on long-term sick leave for burnout: A 3-year follow-up of the REST study. J Rehabil Med [Internet]. 2012;44(8):684–90. Available from: http://www.medicaljournals.se/jrm/content/?doi=10.2340/16501977-1003
45. Ugwoke SC, Eseadi C, Onuigbo LN, Aye EN, Akaneme IN, Oboegbulem AI, et al. A rational-emotive stress management intervention for reducing job burnout and dysfunctional distress among special education teachers. Medicine [Internet]. 2018 Apr 1;97(17):e0475. Available from: https://journals.lww.com/00005792-201804270-00036
46. van der Klink JJL. Reducing long term sickness absence by an activating intervention in adjustment disorders: a cluster randomised controlled design. Occup Environ Med [Internet]. 2003 Jun 1;60(6):429–37. Available from: https://oem.bmj.com/lookup/doi/10.1136/oem.60.6.429
47. van Noppen P, van Dun K, Depestele S, Verstraelen S, Meesen R, Manto M. Transcranial direct current stimulation and attention skills in burnout patients: a randomized blinded sham-controlled pilot study. F1000Res [Internet]. 2020 Feb 14;9:116. Available from: https://f1000research.com/articles/9-116/v1
48. Verkuil B, Brosschot JF, Korrelboom K, Reul-Verlaan R, Thayer JF. Pretreatment of Worry Enhances the Effects of Stress Management Therapy: A Randomized Clinical Trial. Psychother Psychosom [Internet]. 2011 Apr;80(3):189–90. Available from: https://www.karger.com/Article/FullText/320328
49. Willert MV, Thulstrup AM, Bonde JP. Effects of a stress management intervention on absenteeism and return to work – results from a randomized wait-list controlled trial. Scand J Work Environ Health [Internet]. 2011 May;37(3):186–95. Available from: http://www.sjweh.fi/show_abstract.php?abstract_id=3130
50. Willert MV, Thulstrup AM, Hertz J, Bonde JP. Changes in stress and coping from a randomized controlled trial of a three-month stress management intervention. Scand J Work Environ Health [Internet]. 2009 Mar;35(2):145–52. Available from: http://www.sjweh.fi/show_abstract.php?abstract_id=1313
51. Zielhorst T, van den Brule D, Visch V, Melles M, van Tienhoven S, Sinkbaek H, et al. Using a Digital Game for Training Desirable Behavior in Cognitive–Behavioral Therapy of Burnout Syndrome: A Controlled Study. Cyberpsychol Behav Soc Netw [Internet]. 2015 Feb 1;18(2):101–11. Available from: http://www.liebertpub.com/doi/10.1089/cyber.2013.0690
52. Adina M, Vesa Ștefan C, Nirestean A. Burnout Syndrome: Therapeutic Approach With Beneficial Effects on Personality and Quality of Life. Altern Ther Health Med [Internet]. 2021 Nov;27(6):8–14. Available from: http://www.ncbi.nlm.nih.gov/pubmed/33789249
53. Ekstedt M, Söderström M, Åkerstedt T. Sleep physiology in recovery from burnout. Biol Psychol [Internet]. 2009 Dec;82(3):267–73. Available from: https://linkinghub.elsevier.com/retrieve/pii/S030105110900163X

54. Ekvall Hansson E, Håkansson E, Raushed A, Håkansson A. Multidisciplinary program for stress-related disease in primary health care. J Multidiscip Healthc [Internet]. 2009 May;2:61. Available from: http://www.dovepress.com/multidisciplinary-program-for-stress-related-disease-in-primary-health-peer-reviewed-article-JMDH
55. Firth-Cozens J, Hardy GE. Occupational stress, clinical treatment and changes in job perceptions. J Occup Organ Psychol [Internet]. 1992 Jun;65(2):81–8. Available from: https://onlinelibrary.wiley.com/doi/10.1111/j.2044-8325.1992.tb00486.x
56. Grigorescu S, Cazan AM, Rogozea L, Grigorescu DO. Original targeted therapy for the management of the burnout syndrome in nurses: an innovative approach and a new opportunity in the context of predictive, preventive and personalized medicine. EPMA Journal [Internet]. 2020 Jun 6;11(2):161–76. Available from: http://link.springer.com/10.1007/s13167-020-00201-6
57. Kjellgren A, Buhrkall H. Preventing Sick-leave for Sufferers of High Stress-load and Burnout Syndrome: A Pilot Study Combining Psychotherapy and the Flotation tank Torsten Norlander Karolinska Institutet [Internet]. Article in International Journal of Psychology and Psychological Therapy. 2011. Available from: http://www.
58. Lindström C, Åman J, Anderzén-Carlsson A, Lindahl Norberg A. Group intervention for burnout in parents of chronically ill children - a small-scale study. Scand J Caring Sci [Internet]. 2016 Dec 1;30(4):678–86. Available from: https://onlinelibrary.wiley.com/doi/10.1111/scs.12287
59. Meesters Y, Horwitz, van Velzen. Day treatment of patients with severe work-related complaints. Psychol Res Behav Manag [Internet]. 2012 May;5:57. Available from: http://www.dovepress.com/day-treatment-of-patients-with-severe-work-related-complaints-peer-reviewed-article-PRBM
60. Millet P. Integrating Horticulture Into the Vocational Rehabilitation Process of Individuals With Exhaustion Syndrome (Burnout): A Pilot Study. International Journal of Disability Management [Internet]. 2008 Sep 1;3(2):39–53. Available from: https://www.cambridge.org/core/product/identifier/S1833855000000256/type/journal_article
61. Mommersteeg PMC, Keijsers GPJ, Heijnen CJ, Verbraak MJPM, van Doornen LJP. Cortisol deviations in people with burnout before and after psychotherapy: A pilot study. Health Psychology [Internet]. 2006 Mar;25(2):243–8. Available from: http://doi.apa.org/getdoi.cfm?doi=10.1037/0278-6133.25.2.243
62. Mommersteeg PMC, Heijnen CJ, Verbraak MJPM, van Doornen LJP. A longitudinal study on cortisol and complaint reduction in burnout. Psychoneuroendocrinology [Internet]. 2006 Aug;31(7):793–804. Available from: https://linkinghub.elsevier.com/retrieve/pii/S0306453006000527
63. Orosz A, Federspiel A, Eckert A, Seeher C, Dierks T, Tschitsaz A, et al. Exploring the effectiveness of a specialized therapy programme for burnout using subjective report and biomarkers of stress. Clin Psychol Psychother [Internet]. 2021 Jul 15;28(4):852–61. Available from: https://onlinelibrary.wiley.com/doi/10.1002/cpp.2539
64. Pálsdóttir AM, Grahn P, Persson D. Changes in experienced value of everyday occupations after nature-based vocational rehabilitation. Scand J Occup Ther [Internet]. 2013 Sep 17;21(1):1–11. Available from: http://www.tandfonline.com/doi/full/10.3109/11038128.2013.832794

65. Sahlin E, Ahlborg G, Matuszczyk J, Grahn P. Nature-Based Stress Management Course for Individuals at Risk of Adverse Health Effects from Work-Related Stress—Effects on Stress Related Symptoms, Workability and Sick Leave. Int J Environ Res Public Health [Internet]. 2014 Jun 23;11(6):6586–611. Available from: http://www.mdpi.com/1660-4601/11/6/6586
66. Sahlin E, Ahlborg G, Tenenbaum A, Grahn P. Using Nature-Based Rehabilitation to Restart a Stalled Process of Rehabilitation in Individuals with Stress-Related Mental Illness. Int J Environ Res Public Health [Internet]. 2015 Feb 9;12(2):1928–51. Available from: http://www.mdpi.com/1660-4601/12/2/1928
67. Steensma H, Heijer M den, Stallen V. Research Note: Effects of Resilience Training on the Reduction of Stress and Depression among Dutch Workers. Int Q Community Health Educ [Internet]. 2007 Jul 25;27(2):145–59. Available from: http://journals.sagepub.com/doi/10.2190/IQ.27.2.e
68. Thomas TE, Eyal R, Menchavez F, Mocci T, Goldblatt G, Lanoff J, et al. Reducing Workplace Absenteeism Caused by Work Stress in a Health Maintenance Organization Department of Psychiatry. Perm J [Internet]. 2020 Mar;24(1). Available from: http://www.thepermanentejournal.org/doi/10.7812/TPP/19.027
69. van de Leur JC, Buhrman M, Åhs F, Rozental A, Jansen GB. Standardized multimodal intervention for stress-induced exhaustion disorder: an open trial in a clinical setting. BMC Psychiatry [Internet]. 2020 Dec 5;20(1):526. Available from: https://bmcpsychiatry.biomedcentral.com/articles/10.1186/s12888-020-02907-3
70. Hätinen M, Kinnunen U, Mäkikangas A, Kalimo R, Tolvanen A, Pekkonen M. Burnout during a long-term rehabilitation: comparing low burnout, high burnout – benefited, and high burnout – not benefited trajectories. Anxiety Stress Coping [Internet]. 2009 May;22(3):341–60. Available from: http://www.tandfonline.com/doi/abs/10.1080/10615800802567023
71. Kinnunen SM, Puolakanaho A, Tolvanen A, Mäkikangas A, Lappalainen R. Does mindfulness-, acceptance-, and value-based intervention alleviate burnout?—A person-centered approach. Int J Stress Manag [Internet]. 2019 Feb 1;26(1):89–101. Available from: http://doi.apa.org/getdoi.cfm?doi=10.1037/str0000095
72. van der Meulen RT, Valentin S, Bögels SM, de Bruin EI. Mindfulness and self-compassion as mediators of the Mindful2Work Training on perceived stress and chronic fatigue. Mindfulness (N Y) [Internet]. 2021 Apr 20;12(4):936–46. Available from: http://link.springer.com/10.1007/s12671-020-01557-6
